# Dynamic Tensile Strength of Concrete: A Review of Mechanisms, Test Results, and Applications for Dam Safety

**DOI:** 10.3390/ma18245669

**Published:** 2025-12-17

**Authors:** Anderssen Barbosa dos Santos, Pedro Alexandre Conde Bandini, Rocio Lilen Segura, Patrick Paultre

**Affiliations:** 1 Department of Civil Engineering and Building Engineering, University of Sherbrooke, 2500 Boulevard de l’Université, Sherbrooke, QC J1K 2R1, Canada; anderssen.barbosa.dos.santos@usherbrooke.ca (A.B.d.S.); pedro.bandini@usherbrooke.ca (P.A.C.B.); 2 Department of Civil, Geological and Mining Engineering, Polytechnique Montréal, 2500 Chemin de Polytechnique, Montréal, QC H3T 0A3, Canada; rocio-lilen.segura@polymtl.ca

**Keywords:** dynamic tensile strength, strain rate effect, free water effect, preloading effect, crack propagation, inertia effect, large aggregate, mesoscopic modeling, cyclic load, dam guidelines

## Abstract

This paper provides a comprehensive review of the dynamic tensile behavior of concrete, focusing on its implications for seismic-resistant and impact-prone structures such as dams. The present work distinguishes itself in the following ways: providing the first comprehensive synthesis explicitly focused on large-aggregate dam concrete behavior across the seismic strain rate range ($10^{-4}$ to $10^{-2}$ s^−1^), which is critical yet underrepresented in the existing literature; integrating recent experimental and numerical advances regarding moisture effects, load history, and cyclic loading—factors that are essential for dam safety assessments; and critically evaluating current design guidelines for concrete dams against state-of-the-art research to identify gaps between engineering practice and scientific evidence. Through the extensive synthesis of experimental data, numerical simulations, and existing guidelines, the study examines key factors influencing dynamic tensile strength, including strain rate effects, crack evolution, testing techniques, and material variables such as moisture content, load history, and aggregate size. Experimental results from spall tests, split Hopkinson pressure bar configurations, and cyclic loading protocols are analyzed, revealing dynamic increase factors ranging from 1.1 to over 12, depending on the strain rates, saturation levels, and preloading conditions. The roles of inertial effects, free water (via the Stefan effect), and microstructural heterogeneity in enhancing or diminishing tensile performance are critically evaluated. Numerical models, including finite element, discrete element, and peridynamic approaches, are discussed for their ability to simulate crack propagation, inertia-dominated responses, and moisture interactions. The review identifies and analyzes current design guidelines. Key conclusions emphasize the necessity of integrating moisture content, load history, and mesoscale heterogeneity into dynamic constitutive models, alongside standardized testing protocols to bridge gaps between laboratory data and real-world applications. The findings advocate for updated engineering guidelines that reflect recent advances in rate-dependent fracture mechanics and multi-scale modeling, ensuring safer and more resilient concrete infrastructure under extreme dynamic loads.

## 1. Introduction

Concrete, a cornerstone of modern infrastructure, is widely used in critical structures such as dams, bridges, and protective facilities due to its robust compressive strength and durability. However, its tensile behavior under dynamic loading conditions like earthquakes, blasts, or high-speed impacts remains a complex and critical area of study. Unlike static loading, dynamic forces induce strain rate-dependent responses in concrete, significantly altering its tensile strength, fracture mechanisms, and failure patterns [1]. These dynamic effects are especially consequential for the safety and resilience of massive structures like concrete dams, where seismic or impact loads can precipitate catastrophic failures [2,3].

The dynamic increase factor (DIF), which quantifies the enhancement in tensile strength under rapid loading, has been shown to vary from 1.1 at low seismic strain rates ($10^{-2}$ s^−1^) to over 12 at ultra-high rates (>100 s^−1^) [4,5,6]. Earlier studies on the dynamic tensile strength of concrete began with spall experiments performed by Landon [7] and flexural tests by Teller [8], followed by work from Klettke [9], Goldbeck [10], and Wright [11], who reported dynamic increase factors (DIFs) of 1.1–1.2 from flexural tests at low strain rates. At higher strain rates, Fox [12] reported DIFs ranging from 1.2 to 1.9. This body of work was later reviewed by McHenry [13] and Ashton [14]. Lundeen [15] reported a DIF of 1.75 from split tension tests at a strain rate of $10^{-1}\;s^{-1}$, results that were confirmed by Cowell [16], who also observed a DIF of 1.5 at seismic strain rates (approximately $10^{-2}\;s^{-1}$), consistent with findings from Hatano [17]. One of the most notable contributions to the dynamic tensile strength of dam concrete came from Raphael [2], who, based on dam core tests, concluded that tensile strength values should be increased by 50% when used in seismic load design.

Studies involving experimental [4,5,16,18,19,20,21,22,23,24,25,26,27,28,29,30,31,32,33,34,35,36,37,38,39,40,41,42,43,44,45,46,47,48,49,50,51,52,53,54,55,56,57,58,59,60,61,62,63,64,65,66,67,68,69,70,71,72,73,74,75,76,77,78,79,80,81,82] and numerical research [3,30,35,49,53,83,84,85,86,87,88,89,90,91,92,93,94,95,96,97,98,99,100,101,102,103,104,105,106,107,108,109,110,111,112,113,114,115,116,117,118,119,120,121,122,123,124,125,126,127,128,129,130,131,132,133,134,135,136,137,138] have been performed to unravel the mechanisms governing the dynamic tensile strength of concrete. At high strain rates, significant experimental investigations have been conducted using the split Hopkinson pressure bar (SHPB) in both direct and splitting tension configurations [5,23,26], as well as spalling from end impacts [18,19], close-in explosions [22], and plate impact techniques [25]. Reported DIFs range from about 2 at moderate strain rates (2 s^−1^) to over 12 at very high rates (120–157 s^−1^). Ross [4] reported DIFs up to 6.5 at a strain rate of 17.8 s^−1^, while Mellinger and Birkimer [18] observed average DIFs of 6.3 and peak values reaching 8.1 at 20 s^−1^. The highest DIF value was reported by McVay [22], with a DIF of 7.1 at 157 s^−1^. Additionally, Brara et al. [5] and Forquin et al. [6] demonstrated that the DIF increased from 8 to 12 and from 6 to 8, respectively, when comparing dry to wet concrete under strain rates of approximately $10^{2}$ s^−1^.

To understand and extract insights into the general behavior of concrete in tension under different strain rates, researchers [13,139,140,141,142,143,144,145,146,147,148,149,150,151,152,153,154,155] have collected and compared results from various sources in the literature. Soroushian [156] compared literature results in [16,157,158,159], finding that low-strength and wet concrete exhibited higher DIFs. A literature review by Malvar and Crawford [160] compiled data on the tensile strength of concrete at moderate strain rates [16,17,20,21,161,162,163], with values reaching 100 s^−1^ and DIFs up to 2.1. They also included high-strain-rate tests [4,18,19,22,23,25,26,28,164,165,166,167,168,169], where the strain rates ranged from 10^0^ to 10^2^ s^−1^ and DIFs reached up to 8.1. Another significant contribution was made by Pajak [170], who analyzed the influences of testing techniques, materials, and specimen sizes using data from various sources [4,5,28,29,30,32,35,37,38,39,40,41,42,45,46]. To describe the strain rate dependency of ultra-high-performance concrete (UHPC) in tension, Thomas et al. [153] reviewed thirteen previously published studies [37,45,52,57,58,59,60,61,63,65,66,67,68,69,171,172]. They reported strain rates up to 10^2^ s^−1^ and DIF values up to 6, concluding that UHPC behaves similarly to conventional concrete at low and intermediate strain rates, but exhibits significantly greater sensitivity at high strain rates.

**Figure 1:**
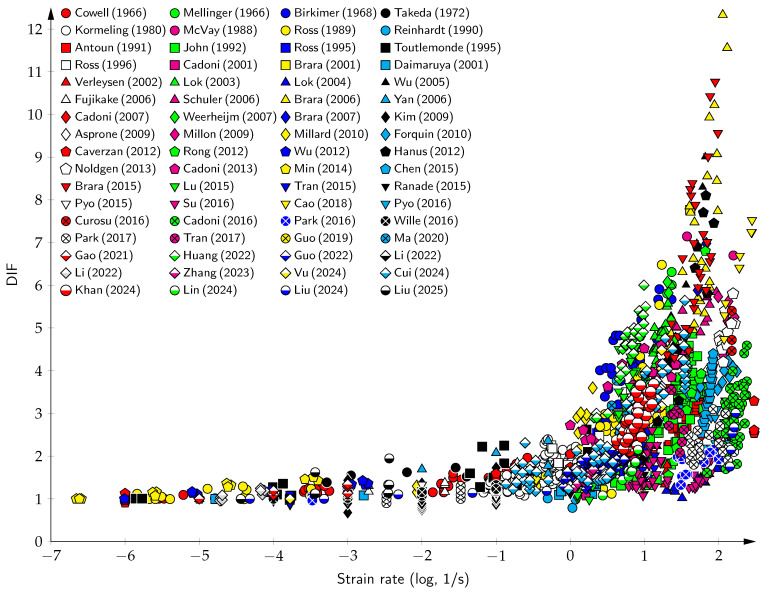
DIF for tensile strength from experimental tests [4,5,16,18,19,20,21,22,23,24,25,26,27,28,29,30,31,32,33,34,35,36,37,38,39,40,41,42,43,44,45,46,47,48,49,50,51,52,53,54,55,56,57,58,59,60,61,62,63,64,65,66,67,68,69,70,71,72,73,74,75,76,77,78,79,80,81,82].

The data from dynamic tension tests, collected from the literature [4,5,16,18,19,20,21,22,23,24,25,26,27,28,29,30,31,32,33,34,35,36,37,38,39,40,41,42,43,44,45,46,47,48,49,50,51,52,53,54,55,56,57,58,59,60,61,62,63,64,65,66,67,68,69,70,71,72,73,74,75,76,77,78,79,80,81,82,82], are presented in Figure 1 as the DIF versus the strain rate. As can be seen, the DIF exhibits sub-horizontal behavior up to a strain rate of 10^1^ s^−1^. In this domain, the viscous effect is dominant, and a single tortuous fracture flattens as the strain rates increase. Above 10^1^ s^−1^, the DIF dramatically shifts to a sub-vertical behavior. In this higher domain, the inertia effect is dominant, and a single crack transitions into multiple macrocracks.

Experimental techniques, ranging from quasi-static servo-hydraulic tests to high-rate split Hopkinson pressure bar (SHPB) methods, have revealed critical insights into crack evolution, inertial effects, and the role of free water through phenomena like the effect of viscous resistance (Stefan effect) [86,173]. Moreover, the interplay between material heterogeneity (e.g., large aggregates in dam concrete) and dynamic loading further complicates predictive modeling and design [174].

Despite advancements, simplified criteria are adopted in guidelines for the dynamic increase factor, particularly for dam safety. For example, while Raphael’s seminal work on dam cores recommends a 50% increase in tensile strength for seismic design [2], contemporary studies emphasize the nuanced effects of saturation, preloading, concrete quality, and grading [47,175,176]. Additionally, numerical simulations have emerged as indispensable tools in capturing mesoscale fracture processes and validating constitutive models, yet challenges remain in reconciling inertial contributions with intrinsic material rate sensitivity [3,177].

This paper synthesizes research on the dynamic tensile strength of concrete into a comprehensive state-of-the-art review, addressing mechanisms, testing methodologies, and practical implications for dam safety. It examines experimental data across strain rate regimes, analyzes crack propagation dynamics, evaluates testing techniques, and critiques DIF formulations. Furthermore, it explores the influences of free water, load history, and large aggregates, while highlighting advancements in numerical modeling and cyclic loading behavior. By integrating these multidisciplinary insights, this review aims to bridge gaps between theoretical research and engineering practice, offering actionable recommendations toward enhancing the seismic resilience and safety of concrete infrastructure.

This paper is organized as follows. First, fundamental mechanisms are explored: crack evolution under dynamic loading (Section 2), experimental testing methodologies (Section 3), and DIF formulations (Section 4). Next, individual influence factors are examined: inertial effects (Section 5), free water and the Stefan effect (Section 6), and load history (Section 7). Material-specific behaviors are then addressed, focusing on large-aggregate dam concrete (Section 8), concrete quality (Section 9), and high-strain-rate responses (Section 10). Advanced topics include numerical modeling approaches (Section 11) and a critical evaluation of design guidelines (Section 12). Finally, a comprehensive discussion of research gaps (Section 13) and conclusions with practical recommendations (Section 14) complete the review.

## 2. Crack Evolution

The fracture behavior of concrete exhibits a significant dependence on the strain rate, with distinct crack initiation, propagation, and failure patterns observed under quasi-static and dynamic loading conditions. At low strain rates from 10^−7^ s^−1^ to 10^−4^ s^−1^, cracks initiate preferentially at interfacial transition zones (ITZs) between aggregates and the matrix, the weakest links in the material system [47,178,179]. Pre-peak sub-critical flaw growth allows cracks to propagate slowly along paths of least resistance, typically circumventing aggregates and following ITZs or weaker matrix regions. This results in rough fracture surfaces and single dominant cracks [36,47,180,181]. Failure occurs at lower stress levels due to localized damage accumulation [47].

At moderate strain rates from 10^−4^ s^−1^ to 10^0^ s^−1^, the viscous resistance of free water in pores further delays crack propagation [47,182,183]. At high strain rates from 10^0^ s^−1^ to 10^4^ s^−1^, rapid stress escalation activates numerous microcracks simultaneously, bypassing weaker interfaces and propagating through stronger aggregate particles [178,179,184]. Inertial forces restrict crack deviation, forcing straighter paths and fracturing tougher aggregates [178,179,180]. This increases fracture energy dissipation and flattens fracture surfaces [47,185]. Multiple cracks coalesce or branch due to stress wave interactions and energy surplus, leading to fragmentation or distributed cracking [79,121,186,187]. The Stefan effect diminishes at very high rates, as water cannot form menisci at crack tips, reducing viscous resistance [188,189,190].

**Figure 2:**
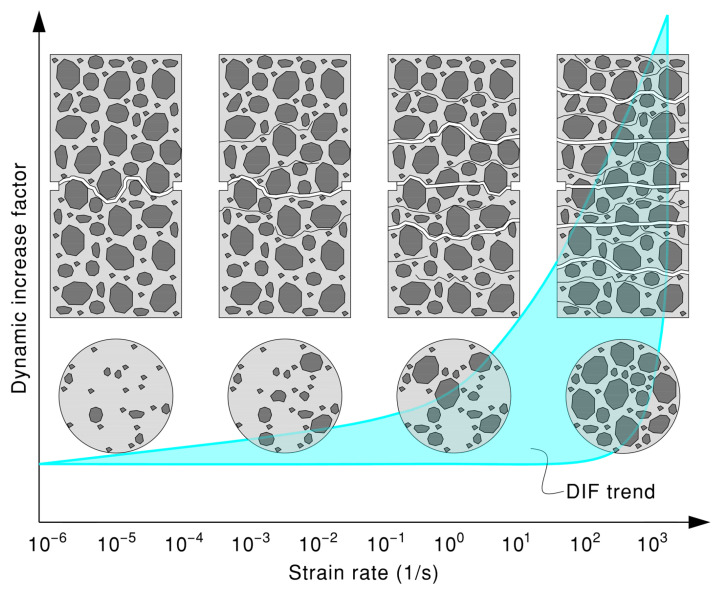
Cracking evolution with an increase in strain rate.

The strain rate dependency of crack evolution, illustrated in Figure 2, arises from time availability, where slow rates allow cracks to navigate weak paths; rapid loading forces shorter, higher-resistance paths [36,181]. The material inertia delays crack initiation, promotes branching, and restricts deflection, increasing aggregate fracture [79,95,179]. The high energy input associated with dynamic loading can induce simultaneous microcracking and aggregate cleavage, enhancing the tensile strength [47,179,191]. The activation of multiple cracks competes with stress wave deactivation, fostering distributed damage [192,193].

In summary, under dynamic tensile loading, concrete’s behavior changes significantly compared to static conditions. The process transitions from failure, governed by the slow, progressive growth of cracks along the weakest paths, to one involving the rapid activation of multiple cracks, extensive branching, and propagation through stronger material [95,107,181,191]. These changes, driven by factors like high strain rates, inertia, and the viscosity of pore water, demand more energy for the fracture process and result in a substantial increase in the apparent tensile strength of concrete [3,75,95].

## 3. Techniques

To ensure the effective analysis and design of both civilian and military structures exposed to extreme conditions—such as blasts, impacts, and shock waves—it is essential to understand how materials behave under a broad spectrum of strain rates, spanning from $10^{-6}$ to $10^{8}$ s^−1^.

Natural phenomena, such as earthquakes, and man-made actions, such as explosions, can generate strain rates that span these domains and significantly influence the dynamic responses of construction materials. To assess such behavior in laboratory conditions, experimental techniques and tools such as servo-hydraulic testing, drop-weight impact systems, the split Hopkinson pressure bar (SHPB), and plate impact tests are commonly employed. Servo-hydraulic machines are ideal for quasi-static and intermediate strain rates from 10^−7^ s^−1^ to 10^1^ s^−1^; drop weight hammers are used for intermediate strain rates spanning 10^−1^ s^−1^ to 10^2^ s^−1^; and the SHPB method [194] is effective for high strain rates up to 10^4^ s^−1^ [140,151,153,170,195]. For very high strain rates, tools such as flyer plates and Taylor anvils are used, although they are less common for concrete due to the limited availability of experimental data [154,170]. Table 1 briefly describes the experimental techniques used for the dynamic testing of concrete [146,151,152,153,154,170,194,196,197,198,199], linking the techniques to the load types and covered strain rate ranges.

**Figure 3:**
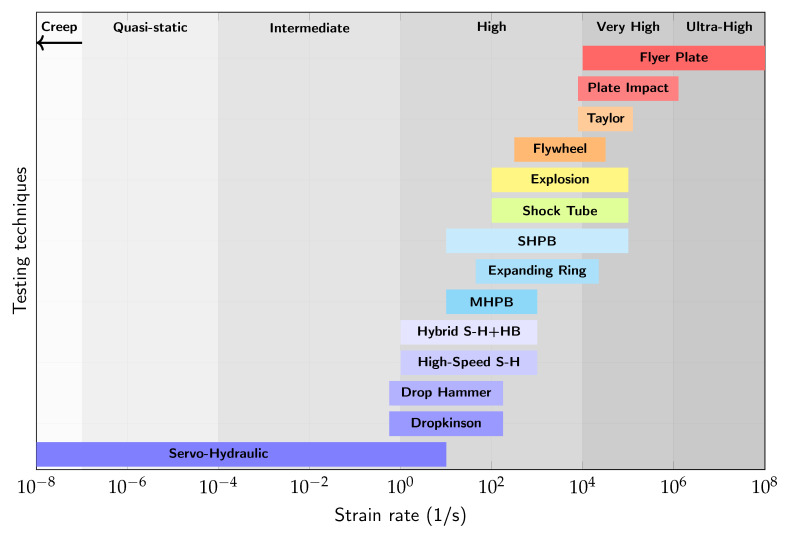
Strain rate domains and their corresponding experimental techniques.

Figure 3 presents the strain rate domains and the experimental methods suited to each range. Strain rates below $10^{-6}$ s^−1^ fall into the creep region, while the interval between $10^{-6}$ and $10^{-1}$ s^−1^ defines the quasi-static domain. The intermediate strain rate zone lies between $10^{0}$ and $10^{2}$ s^−1^, followed by the high strain rate range from $10^{2}$ to $10^{4}$ s^−1^. Strain rates between $10^{4}$ and $10^{6}$ s^−1^ are termed very high, and those exceeding $10^{6}$ s^−1^ fall into the ultra-high strain rate category.

## 4. Dynamic Increase Factor (DIF)

The rate-dependent formulation for the DIF was first introduced by Komlos [200], as summarized in Table 2. Later, Soroushian et al. [156] proposed an empirical equation to predict the strain rate effect on concrete tensile strength using least-squares curve fitting to experimental data.

Mihashi and Izumi [201] suggested that the influence of the loading rate could be represented by a power-law function, derived from a stochastic theory of concrete fracture. This approach was further developed in a subsequent study [202] and later adopted in the CEB-FIP Model Code [203] as a bilinear formulation, which introduces a slope discontinuity at a strain rate of 30 s^−1^.

Building upon early experimental studies [4,16,20,21,161,163], which were extended up to 1 s^−1^, and additional datasets covering higher rates up to 200 s^−1^ [4,18,19,22,25,26], Malvar and Ross [155] proposed a modification to the CEB formulation. Their analysis supported a transition strain rate at 1 s^−1^, rather than the 30 s^−1^ assumed in the original model. Their revised model is also presented in Table 2. More recently, Pajak [170] compiled updated test data on concrete under varying tensile strain rates and concluded that the transition strain rate cannot be definitively identified, in contrast to the assumptions of both the CEB [203] and Malvar and Ross [155] models. Instead, Pajak proposed that a continuous curve better represents the strain rate effect across a broad range. This continuous model was later formulated by Hong and Kang [152], as also shown in Table 2.

In recent years, constitutive models have been developed [145,153,204,205], incorporating updated experimental findings and supported by numerical simulations that help to isolate and explain the mechanisms contributing to the dynamic response of concrete. A comprehensive overview of the DIF formulations is provided in Table 2.

**Table 2:** Strain rate-dependent DIF formulations.

Author	Dynamic Increase Factor	Applicability	Parameters
1. Simple Logarithmic Models			
1.1 Linear Logarithmic			
[200]	$1.0+0.1\times \log\left(\frac{\dot{\varepsilon }}{\dot{\varepsilon_{s}}}\right)$	$\dot{\varepsilon }\leq 1\;s^{-1}$	${\dot{\varepsilon }}_{s}=1\times 10^{-6}\;s^{-1}$
			Dynamic strain rate, $\dot{\varepsilon }$
[36]	$1.0+0.135\times \log\left(\frac{\dot{\varepsilon }}{{\dot{\varepsilon }}_{s}}\right)$	$\dot{\varepsilon }\leq 1\;s^{-1}$	${\dot{\varepsilon }}_{s}=1\times 10^{-5}\;s^{-1}$
			Dynamic strain rate, $\dot{\varepsilon }$
[36]	$1.0+0.265\times \log\left(\frac{\dot{\varepsilon }}{{\dot{\varepsilon }}_{s}}\right)$	$\dot{\varepsilon }\leq 1\;s^{-1}$	${\dot{\varepsilon }}_{s}=1\times 10^{-5}\;s^{-1}$
		Wet concrete	Dynamic strain rate, $\dot{\varepsilon }$
[206]	$1.0+0.1853\times \log\left(\frac{\dot{\varepsilon }}{{\dot{\varepsilon }}_{s}}\right)\;$	$\dot{\varepsilon }\leq 1\;s^{-1}$	${\dot{\varepsilon }}_{s}=1\times 10^{-6}\;s^{-1}$
			Dynamic strain rate, $\dot{\varepsilon }$
1.2 Quadratic Logarithmic			
[156]	$1.77+0.219\times \log\;\dot{\varepsilon }+0.0154{\left(\log\;\dot{\varepsilon }\right)}^{2}$	$\dot{\varepsilon }\leq 1\;s^{-1}$	Dynamic strain rate, $\dot{\varepsilon }$
[207]	$1.0+0.1948\times \log\left(\frac{\dot{\varepsilon }}{{\dot{\varepsilon }}_{s}}\right)+0.03583\times \left[\log{\left(\frac{\dot{\varepsilon }}{{\dot{\varepsilon }}_{s}}\right)}^{2}\right]$	$\dot{\varepsilon }\leq 1\;s^{-1}$	${\dot{\varepsilon }}_{s}=1\times 10^{-6}\;s^{-1}$
			Dynamic strain rate, $\dot{\varepsilon }$
[208]	$0.8267-0.02987\times \log\dot{\varepsilon }+0.04379\times {\left(\log\dot{\varepsilon }\right)}^{2}$	$\dot{\varepsilon }\leq 1\;s^{-1}$	Dynamic strain rate, $\dot{\varepsilon }$
2. Power-Law Models			
2.1 Simple Power Law			
[24]	${\left(\frac{\dot{\varepsilon }}{{\dot{\varepsilon }}_{s}}\right)}^{n}$	$\dot{\varepsilon }\leq 1\;s^{-1}$	$n=1/(10+\left(f_{cs}/2\right))$
			Dynamic strain rate, $\dot{\varepsilon }$
			Compressive strength (MPa), $f_{cs}$
[209]	${\left(\frac{\dot{\varepsilon }}{{\dot{\varepsilon }}_{s}}\right)}^{0.081}\;$	$\dot{\varepsilon }\leq 1\;s^{-1}$	${\dot{\varepsilon }}_{s}=1\times 10^{-5}\;s^{-1}$
			Dynamic strain rate, $\dot{\varepsilon }$
2.2 Variable Exponent Power Law			
[23]	$e^{\left(0.00126\times {\left(\log\left(\frac{\dot{\varepsilon }}{{\dot{\varepsilon }}_{s}}\right)\right)}^{3.373}\right)}\;$		${\dot{\varepsilon }}_{s}=1\times 10^{-7}\;s^{-1}$
			Dynamic strain rate, $\dot{\varepsilon }$
[210]	${\left(\frac{\dot{\varepsilon }}{{\dot{\varepsilon }}_{s}}\right)}^{\left(0.0013\times {\left(\log\left(\frac{\dot{\varepsilon }}{{\dot{\varepsilon }}_{s}}\right)\right)}^{1.95}\right)}$		${\dot{\varepsilon }}_{s}=1\times 10^{-6}\;s^{-1}$
			Dynamic strain rate, $\dot{\varepsilon }$
[211]	${\left(\frac{\dot{\varepsilon }}{{\dot{\varepsilon }}_{s}}\right)}^{0.002\times {\left(\mathrm{HSV}\right)}^{0.19}}$	$\dot{\varepsilon }\leq 1\;s^{-1}$	${\dot{\varepsilon }}_{s}=1\times 10^{-6}s^{-1}$
			Dynamic strain rate, $\dot{\varepsilon }$
			HSV (DT) = $\pi D^{2}L/4$
			HSV (MOR) = 14bhl/1600
			*D* = diameter of specimen (mm)
			*L* = length of specimen (mm)
			*b* = width of specimen (mm)
			*h* = height of specimen (mm)
			*l* = span length of specimen (mm)
3. Bilinear/Piecewise Formulations			
[203]	${\left(\frac{\dot{\varepsilon }}{{\dot{\varepsilon }}_{s}}\right)}^{1.016\times \delta_{s}}$	$\dot{\varepsilon }\leq 30\;s^{-1}$	${\dot{\varepsilon }}_{s}=1\times 10^{-6}\;s^{-1}$
	$\beta_{s}\times {\left(\frac{\dot{\varepsilon }}{{\dot{\varepsilon }}_{s}}\right)}^{1/3}$	$\dot{\varepsilon }>30\;s^{-1}$	$\log\beta_{s}=7.112\times \delta_{s}-2.33$
			$\delta =1/(10+6\times f_{cs}/f_{c0})$
			$f_{c0}$ = 10 (MPa)
			Dynamic strain rate, $\dot{\varepsilon }$
			Compressive strength (MPa), $f_{cs}$
[155,160]	${\left(\frac{\dot{\varepsilon }}{{\dot{\varepsilon }}_{s}}\right)}^{\delta_{s}}$	$\dot{\varepsilon }\leq 1\;s^{-1}$	${\dot{\varepsilon }}_{s}=10^{-6}\;s^{-1}$
	$\beta_{s}\times {\left(\frac{\dot{\varepsilon }}{{\dot{\varepsilon }}_{s}}\right)}^{1/3}$	$\dot{\varepsilon }>1\;s^{-1}$	$\log\beta_{s}=6\times \delta_{s}-2$
			$\delta =1/(1+8\times f_{cs}/f_{co})$
			$f_{c0}$ = 10 (MPa)
			Dynamic strain rate, $\dot{\varepsilon }$
			Compressive strength (MPa), $f_{cs}$
[212]	${\left(\frac{\dot{\varepsilon }}{{\dot{\varepsilon }}_{s}}\right)}^{0.018}$	$\dot{\varepsilon }\leq 10\;s^{-1}$	${\dot{\varepsilon }}_{s}=1.0\times 10^{-6}\;s^{-1}$
	$0.0062{\left(\frac{\dot{\varepsilon }}{{\dot{\varepsilon }}_{s}}\right)}^{1/3}$	$10\leq \dot{\varepsilon }\leq 300\;s^{-1}$	Dynamic strain rate, $\dot{\varepsilon }$
[213]	$1.78+0.13\times \log\left(\frac{\dot{\varepsilon }}{{\dot{\varepsilon }}_{s}}\right)$	$\dot{\varepsilon }\leq 2\;s^{-1}$	${\dot{\varepsilon }}_{s}=1.0\times 10^{-5}\;s^{-1}$
	$0.23+1.45\ln\left(1+\frac{\dot{\varepsilon }}{{\dot{\varepsilon }}_{s}}\right)$	$\dot{\varepsilon }>2\;s^{-1}$	Dynamic strain rate, $\dot{\varepsilon }$
[66]	${\left(\frac{\dot{\varepsilon }}{{\dot{\varepsilon }}_{s}}\right)}^{0.01465}$	$\dot{\varepsilon }\leq 25\;s^{-1}$	${\dot{\varepsilon }}_{s}=3.33\times 10^{-6}s^{-1}$
	$0.002352{\left(\frac{\dot{\varepsilon }}{{\dot{\varepsilon }}_{s}}\right)}^{0.3735}$	$\dot{\varepsilon }>25\;s^{-1}$	Dynamic strain rate, $\dot{\varepsilon }$
[153]	${\left(\frac{\dot{\varepsilon }}{{\dot{\varepsilon }}_{s}}\right)}^{\delta }$	$\dot{\varepsilon }\leq 10\;s^{-1}$	${\dot{\varepsilon }}_{s}=1\times 10^{-6}s^{-1}$
	$\beta {\left(\frac{\dot{\varepsilon }}{{\dot{\varepsilon }}_{s}}\right)}^{3/4}$	$\dot{\varepsilon }>10\;s^{-1}$	$\delta =\frac{1}{1+8\frac{f_{cs}}{f_{c0}}}$
			logβ=7δ−5.25
			$f_{c0}$ = 10 (MPa)
			Dynamic strain rate, $\dot{\varepsilon }$
			Compressive strength (MPa), $f_{cs}$
[153]	${\left(\frac{\dot{\varepsilon }}{{\dot{\varepsilon }}_{0}}\right)}^{1/121}$	$\dot{\varepsilon }\leq 1\;s^{-1}$	${\dot{\varepsilon }}_{s}=1\times 10^{-6}s^{-1}$
	$\beta {\left(\frac{\dot{\varepsilon }}{{\dot{\varepsilon }}_{0}}\right)}^{3/4}$	$\dot{\varepsilon }>1\;s^{-1}$	$\delta =\frac{1}{1+8\frac{f_{cs}}{f_{c0}}}$
		High-strength concrete	logβ=6δ−4.5
			$f_{c0}$ = 10 (MPa)
			Dynamic strain rate, $\dot{\varepsilon }$
			Compressive strength (MPa), $f_{cs}$
[68]	${\left(\frac{\dot{\varepsilon }}{{\dot{\varepsilon }}_{s}}\right)}^{\delta }$	$\dot{\varepsilon }\leq 25\;s^{-1}$	${\dot{\varepsilon }}_{s}$ = $3.33\times 10^{-4}\;s^{-1}$
	$\beta {\left(\frac{\dot{\varepsilon }}{{\dot{\varepsilon }}_{s}}\right)}^{\eta }$	$\dot{\varepsilon }>25\;s^{-1}$	$\delta =0.017-2722\times {\left(\frac{f_{cs}}{f_{c0}}\right)}^{-7.33}$
			$\log\beta =-0.007082\times (-2.08f_{cs})$
			$\eta =0.1208\times f_{cs}^{0.2622}$
			$f_{c0}$ = 10 (MPa)
			Dynamic strain rate, $\dot{\varepsilon }$
			Compressive strength (MPa), $f_{cs}$
[205]	${\left(\frac{\dot{\varepsilon }}{{\dot{\varepsilon }}_{s}}\right)}^{0.0315}$	$\dot{\varepsilon }<0.54\;s^{-1}$	${\dot{\varepsilon }}_{s}=1\times 10^{-6}\;s^{-1}$
	$0.000682{\left(\frac{\dot{\varepsilon }}{{\dot{\varepsilon }}_{s}}\right)}^{0.583}$	$\dot{\varepsilon }\geq 0.54\;s^{-1}$	Dynamic strain rate, $\dot{\varepsilon }$
[205]	${\left(\frac{\dot{\varepsilon }}{{\dot{\varepsilon }}_{s}}\right)}^{0.0133}$	$\dot{\varepsilon }<0.21\;s^{-1}$	${\dot{\varepsilon }}_{s}=1\times 10^{-6}\;s^{-1}$
	$0.04953{\left(\frac{\dot{\varepsilon }}{{\dot{\varepsilon }}_{s}}\right)}^{0.259}$	$\dot{\varepsilon }\geq 0.21\;s^{-1}$	Dynamic strain rate, $\dot{\varepsilon }$
4. Fiber-Reinforced Concrete Models			
[214]	${\left(\frac{\dot{\varepsilon }}{{\dot{\varepsilon }}_{s}}\right)}^{h\delta }$	$\dot{\varepsilon }\leq 1\;s^{-1}$	${\dot{\varepsilon }}_{s}=1\times 10^{-6}s^{-1}$
	$\beta {\left(\frac{\dot{\varepsilon }}{{\dot{\varepsilon }}_{s}}\right)}^{k/3}$	$\dot{\varepsilon }>1\;s^{-1}$	logβ=6hδ−2
			$\delta =\frac{1}{1+8m\frac{f_{cs}}{f_{c0}}}$
			$f_{c0}$ = 10 (MPa)
			m=0.75
			h=1,k=1, hooked fiber
			h=1.3,k=0.8, twisted fiber
			Dynamic strain rate, $\dot{\varepsilon }$
			Compressive strength (MPa), $f_{cs}$
[215]	$3.0701\log\dot{\varepsilon }+0.7085$	$2\leq \dot{\varepsilon }\leq 20\;s^{-1}$ Plain concrete	Dynamic strain rate, $\dot{\varepsilon }$
	$3.4471\log\dot{\varepsilon }+1.3493$	$2\leq \dot{\varepsilon }\leq 20\;s^{-1}$ 0.5 % steel fiber	
	$3.1296\log\dot{\varepsilon }+2.2996$	$2\leq \dot{\varepsilon }\leq 20\;s^{-1}$ 1.0 % steel fiber	
	$4.0106\log\dot{\varepsilon }+2.3491$	$2\leq \dot{\varepsilon }\leq 20\;s^{-1}$ 1.5 % steel fiber	
[216]	${\left(\frac{\dot{\varepsilon }}{{\dot{\varepsilon }}_{s}}\right)}^{k\delta }$	$\dot{\varepsilon }\leq {\dot{\varepsilon }}_{t}\;s^{-1}$	${\dot{\varepsilon }}_{s}=1\times 10^{-6}s^{-1}$
	$m\beta {\left(\frac{\dot{\varepsilon }}{{\dot{\varepsilon }}_{s}}\right)}^{1/3}$	$\dot{\varepsilon }>{\dot{\varepsilon }}_{t}\;s^{-1}$	${\dot{\varepsilon }}_{t}=2-20\;s^{-1}$
			$\delta =\frac{1}{1+8\frac{f_{cs}}{f_{c0}}}$
			logβ=7kδ−2.141
			$f_{c0}$ = 10 (MPa)
			*k* = 0.8, straight fiber
			*k* = 0.95, hooked fiber
			*k* = 1.3, twisted fiber
			Dynamic strain rate, $\dot{\varepsilon }$
			Compressive strength (MPa), $f_{cs}$
			Fiber volume fraction, *m*
[217]	$0.0095\dot{\varepsilon }+1.0345$	$\dot{\varepsilon }\leq 30\;s^{-1}$	Dynamic strain rate, $\dot{\varepsilon }$
	$0.00004{\dot{\varepsilon }}^{2}-0.0436\dot{\varepsilon }+0.2981$	$\dot{\varepsilon }>30\;s^{-1}$	
		Concrete with fiber	
[145]	$0.0508\log(\dot{\varepsilon })+1.3049$	$\dot{\varepsilon }<10\;s^{-1}$	Dynamic strain rate, $\dot{\varepsilon }$
	$1.7139\log(\dot{\varepsilon })-0.4846$	$\dot{\varepsilon }\geq 10\;s^{-1}$	
		Concrete with fiber	
5. Piecewise Linear/Polynomial Models			
[218]	$1.833+0.1425\times \log\times \dot{\varepsilon }\geq 1.0$	$\dot{\varepsilon }>2.32\;s^{-1}$	Dynamic strain rate, $\dot{\varepsilon }$
	$0.814+2.929\times \log\times \dot{\varepsilon }\leq 6.0$	$\dot{\varepsilon }\leq 2.32\;s^{-1}$	
[219]	$1.12+0.0225\times \log\dot{\varepsilon }$	$\dot{\varepsilon }\leq 0.1\;s^{-1}$	Dynamic strain rate, $\dot{\varepsilon }$
	$1.6+1.235\times \log\dot{\varepsilon }+0.73625\times {\left(\log\dot{\varepsilon }\right)}^{2}$	$0.1\leq \dot{\varepsilon }\leq 50\;s^{-1}$	
[187]	$2.06+0.26\times \log\dot{\varepsilon }$	$\dot{\varepsilon }\leq 1\;s^{-1}$	Dynamic strain rate, $\dot{\varepsilon }$
	$2.06+2\times \log\dot{\varepsilon }$	$\dot{\varepsilon }>1\;s^{-1}$	
[170]	$1.5\times {\dot{\varepsilon }}^{0.036}$	$\dot{\varepsilon }<0.1\;s^{-1}$	Dynamic strain rate, $\dot{\varepsilon }$
	$1.84\times {\dot{\varepsilon }}^{0.12}$	$0.1\leq \dot{\varepsilon }<1\;s^{-1}$	
	$1.7+0.2\times \dot{\varepsilon }-0.0067\times {\dot{\varepsilon }}^{2}$	$1\leq \dot{\varepsilon }<10\;s^{-1}$	
	$2.34+0.063\times \dot{\varepsilon }+0.0004\times {\dot{\varepsilon }}^{2}$	$10\;s^{-1}\leq \dot{\varepsilon }$	
[217]	$0.0215\dot{\varepsilon }+0.916$	$\dot{\varepsilon }\leq 30\;s^{-1}$	Dynamic strain rate, $\dot{\varepsilon }$
	$0.0256\dot{\varepsilon }+0.6867$	$\dot{\varepsilon }>30\;s^{-1}$	
[220]	$0.1424\log\dot{\varepsilon }+1.833$	$<2.32\;s^{-1}$	Dynamic strain rate, $\dot{\varepsilon }$
	$2.92\log\dot{\varepsilon }+0.814$	$>2.32\;s^{-1}$	
6. Direct Power-Law Models			
[221]	$1+1.505{\dot{\varepsilon }}^{0.295}$	${\dot{\varepsilon }}_{s}>1\times 10^{-6}\;s^{-1}$	Dynamic strain rate, $\dot{\varepsilon }$
[222]	$1.51\times {\dot{\varepsilon }}^{0.03}$	$\dot{\varepsilon }\geq 1\;s^{-1}$	Dynamic strain rate, $\dot{\varepsilon }$
	$1.52\times {\dot{\varepsilon }}^{0.33}$	$\dot{\varepsilon }<1\;s^{-1}$	
[204]	$1+{\left(\frac{\dot{\varepsilon }}{5}\right)}^{0.876}$	${\dot{\varepsilon }}_{s}>1\times 10^{-6}\;s^{-1}$	Dynamic strain rate, $\dot{\varepsilon }$
7. Complex/Special Models			
[223]	$1.95-3.32\times \left(\frac{1-{\dot{\varepsilon }}^{1/8}}{2.2+3.2\times {\dot{\varepsilon }}^{1/8}}\right)$	$\dot{\varepsilon }\leq 1\;s^{-1}$	Dynamic strain rate, $\dot{\varepsilon }$
[224]	$\left\{\tanh\left[\left(\log\left(\frac{\dot{\varepsilon }}{{\dot{\varepsilon }}_{0}}\right)-W_{s}\right)\times S\right]\times \left[\frac{F_{m}}{W_{y}}-1\right]-1\right\}$		${\dot{\varepsilon }}_{0}=1\times 1.0\;s^{-1}$ $F_{m}$ is the limit enhancement $W_{x},\;S\;\mathrm{and}\;W_{y}$ are the curve fitting parameters
[225]	$0.99679+0.1536\times n+0.02326\times \log\left(\frac{\dot{\varepsilon }}{{\dot{\varepsilon }}_{s}}\right)$	$\dot{\varepsilon }\leq 1\;s^{-1}$	*n* = axial compression ratio ${\dot{\varepsilon }}_{s}=1\times 10^{-5}\;s^{-1}$ Dynamic strain rate, $\dot{\varepsilon }$

## 5. Inertia Effects

Studies have demonstrated that the apparent increase in the dynamic tensile strength of concrete at high strain rates is influenced not only by strain rate sensitivity but also by inertial effects, particularly axial and lateral inertial forces [3,110,177,226,227,228]. Researchers emphasize that inertial forces—both axial and lateral—can significantly increase the apparent strength in tests such as the split Hopkinson pressure bar (SHPB) test and Brazilian splitting. When the strain rates exceed approximately 1 s^−1^, wave propagation, stress confinement, and accelerated crack growth (including branching) cause temporary resistance, elevating the measured strength. Additionally, the specimen geometry, density, and local hydrostatic pressure influence the magnitudes of these inertial contributions. Cotsovos [228] attributes strength increases primarily to inertia effects under high-strain-rate tension, suggesting that geometric factors, stress-wave travel distances, and localized failure behavior dominate. Meanwhile, Lu et al. [229] describe a micromechanism in which inertia at the microcrack level delays crack propagation, implying that the strain rate effect is, in part, an inherent material phenomenon rather than purely structural.

Numerical and experimental investigations demonstrate that the measured dynamic tensile strength of concrete—often termed the apparent strength—includes a significant contribution from inertial forces. The apparent stress ($\sigma_{\mathrm{apparent}}$) can be decomposed into the effective material stress ($\sigma_{\mathrm{effective}}$) and an inertial component ($\sigma_{\mathrm{inertia}}$), allowing the latter to be isolated through modeling approaches. A common quantification strategy involves employing strain rate-insensitive constitutive models, where any strength enhancement under dynamic loading conditions originates purely from inertia confinement. Using this approach, studies [110,230,231] have defined an inertial dynamic increase factor (DIF_i_), which, when subtracted from the experimentally observed apparent DIF_a_, yields the intrinsic material strain rate component (${\mathrm{DIF}}_{\dot{\varepsilon }}$). For instance, simulations with rate-insensitive material models have revealed that the tensile strength could increase up to fourfold at a strain rate of 120 s^−1^ due solely to inertial confinement, indicating the substantial impact of inertia in dynamic Brazilian disk tests [110]. An equivalent momentum scheme (EMS) [3] further quantified the total dynamic enhancement by separating it into an inertia effect factor (DIF_inertia_) and a multiple crack factor (DIF_crack_), with the former found to be three to five times larger than the latter. In mesoscale simulations, the inertial stress was also shown to contribute 23–39% of the apparent strength depending on the specimen size, confirming inertia’s dominant and quantifiable role in dynamic tensile strength enhancement [3,177,232,233].

The magnitude of the inertial influence in dynamic tensile tests is intrinsically linked to the specimen geometry, mass, and loading rate. Larger specimens possess greater inertia and thus exhibit higher apparent dynamic tensile strength. Unlike the static size effect, where the strength typically decreases with increasing specimen dimensions, under dynamic loading—particularly above a critical strain rate of approximately 1 s^−1^—larger specimens often display higher dynamic tensile strength [3,79,100,114,116,234,235]. This behavior arises because the vertical and lateral inertia confinement intensify with the specimen size, amplifying the apparent DIF. Experimental studies show that thin discs in Brazilian tests, or tubes with large inner diameters, can experience significant lateral inertia confinement, leading to high tensile strength [110,112]. Numerical studies have shown that, as the strain rate increases, the lateral inertia confinement progressively dominates the response; for strain rates below the critical threshold, size effects are diminished, while, beyond it, inertia confinement stabilizes the tensile response and can even eliminate or invert traditional size effect trends [116]. In thicker or larger specimens, inertia can be even stronger since mass and confinement effects are greater—an observation that can reverse the classic quasi-static size effect, in which smaller specimens are normally considered stronger [3,177,227].

Notably, Pan et al. [3] identify a threshold strain rate range of approximately 0.01 s^−1^, beyond which inertia dominates strength enhancement. The inertia effect is the underlying cause of the dynamic size effect, and the equivalent inertia strength increases with increasing sizes above the threshold between 2^−3^ and 2 s^−1^. Consequently, the dynamic size effect is not a material property but a structural manifestation governed by the specimen geometry, the strain rate, and the spatial distribution of inertial stresses within the loading configuration [3,110,116,230].

On the other hand, other studies [120,229] have suggested that genuine material strain rate sensitivity could be the primary reason for the observed increase in tensile strength. Their numerical simulations, designed to exclude any true rate-dependent parameters, indicate that purely inertial effects alone lead to only modest strength increases, underscoring the necessity of intrinsic rate-dependent constitutive behavior.

Overall, research shows that both strain rate sensitivity and inertia increase concrete’s dynamic tensile strength. While moderate strain rates reflect genuine material behavior, at higher rates, inertia dominates, increasing the measured strength.

## 6. Influence of Free Water

The influence of free water on the dynamic tensile strength of concrete has been studied through both experimental and theoretical research to reveal the interplay between the material composition, pore structure, and loading conditions. At quasi-static strain rates, saturated concrete exhibits reduced tensile strength compared to dry specimens due to the lubricating effect of free water, which weakens particle bonds and promotes crack propagation [156,236,237]. However, under dynamic loading, saturated concrete demonstrates significantly higher tensile strength, with the DIF reaching values exceeding 10 at strain rates above 100 s^−1^ [5,55,86,238].

**Figure 4:**
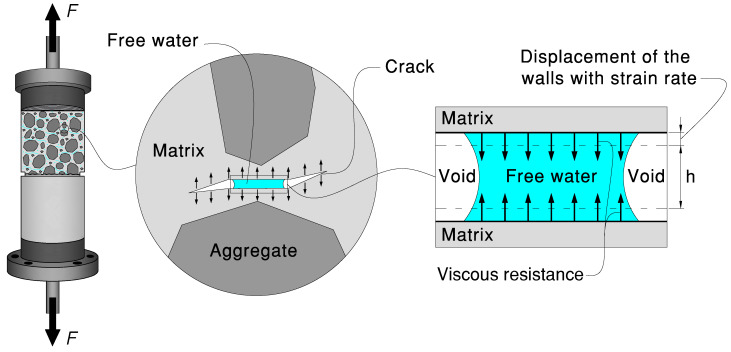
Multiscale illustration of the Stefan effect in concrete under dynamic tensile loading.

This behavior can be explained by the Stefan effect [239], where a viscous resistance mechanism arises from water trapped in nanopores and microcracks during rapid deformation (see Figure 4). As cracks initiate and propagate, the movement of free water generates cohesive stresses that oppose crack opening, delaying fracture localization and enhancing the tensile strength [173,190,233,240,241,242]. The Stefan effect is particularly pronounced in concretes with higher porosity, where greater water absorption amplifies viscous drag forces [77,163,243,244].

Experimental studies using split Hopkinson pressure bar (SHPB), spalling, and impact tests have validated this phenomenon across a wide strain rate spectrum ($10^{-6}$ to $10^{3}$ s^−1^). Reinhardt [24] showed that wet microconcrete samples exhibited 200–300% strength gains over dry ones at strain rates of 1–10 s^−1^. Similarly, Rossi et al. [173] observed DIF values greater than 4 in saturated concrete, far exceeding the 1.6 of dry concrete. These results were further supported by Brara and Klepaczko [5] and Erzar and Forquin [86], who observed strength gains of 7–12 times in wet specimens.

Advanced imaging tools such as high-speed cameras and digital image correlation (DIC) have confirmed that free water delays crack initiation, expanding the fracture process zone and increasing energy dissipation [77,238]. This delay is crucial in explaining the increased toughness of wet concrete under fast loading conditions.

A transition strain rate can be identified around 1 s^−1^, marking a reduction in the effect of viscous resistance (Stefan effect) and the onset of inertial forces. Below this threshold, the tensile strength increases linearly with the logarithm of the strain rate [182,245]. Above ∼10 s^−1^, inertial effects dominate, driving cracks to propagate through aggregates rather than interfacial zones, thereby homogenizing the failure path [6,30,41]. Even in this high-rate regime, saturated concrete maintains an advantage: the residual pore pressure, hydraulic damping, and delayed crack propagation continue to enhance the DIF values [5,78,82].

The material composition and pore structure further shape the moisture influence. High *w/c* ratio concretes with more pores absorb more water, amplifying the Stefan effect at the cost of lower static strength [47,163,243,244]. In contrast, low-porosity, high-strength concretes (HSCs) exhibit reduced rate sensitivity due to limited water availability [78,188]. Interestingly, fiber-reinforced composites like ultra-high-toughness cementitious composites (UHTCCs) demonstrate intermediate behavior; fibers help to bridge cracks and reduce stress concentration, but they also suppress water’s viscous contribution, yielding lower DIF gains than in plain concrete [82,188].

Modeling approaches have been aligned with experimental results. Rossi [246] proposed viscoelastic damage models that embed water’s resistance as a rate-dependent hardening parameter. Brara and Klepaczko [5] implemented the Stefan effect in cumulative damage laws validated by spall and FEM simulations. Selyutina and Petrov [247] introduced a fracture incubation time model, linking the strength increase to the energy dissipation delay caused by water in pores. More recent works [238,248] suggest the need for DIF equations that include saturation.

From an engineering perspective, this research is crucial. In blast-resistant, seismic, or impact-prone structures, leveraging water’s role through tailored porosity and optimized *w/c* ratios can enhance performance without compromising static durability [77,238]. There are still important gaps, such as the underexplored seismic strain rate range [55]. Multiaxial loading, long-term wetting–drying cycles, and fiber–water interactions require further exploration [29,248].

**Figure 5:**
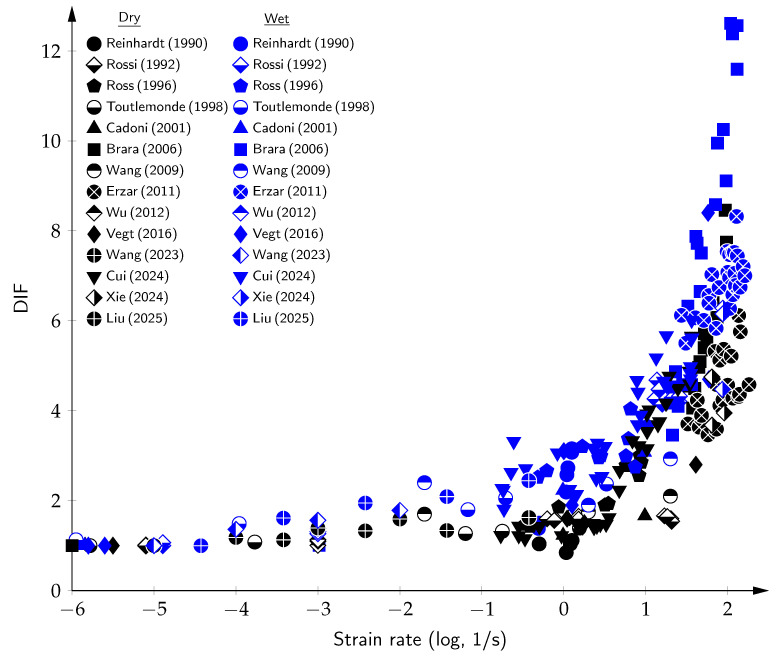
Results from experimental tension tests on concrete under wet and dry conditions [5,6,24,28,29,47,78,82,173,190,236,238,244,248].

A quantitative evaluation is shown in Table 3, which compares DIFs from experimental studies comparing wet and dry concrete results. In summary, free water enhances the dynamic tensile strength of concrete through mechanisms that vary with the strain rate and material composition. While moisture reduces the quasi-static strength, it enhances the dynamic performance via viscous, pore pressure, and inertial effects. This enhancement can be observed in terms of the DIF in Figure 5, where the results from experimental tension tests on concrete under wet and dry conditions are compared.

## 7. Influence of Load History

### 7.1. Monotonic Loading

Understanding the mechanical behavior of concrete under dynamic loads has become increasingly crucial in the design of seismic-resistant infrastructure, particularly in the context of massive structures like dams. A key variable influencing tensile performance under such conditions is the load history—specifically, the presence of initial static stress or preload prior to dynamic excitation.

Across the experimental investigations, a consistent trend emerges: initial static loading has a non-negligible and often nonlinear effect on the tensile strength of concrete [47,54,175,206,249,250,251,252,253]. This influence varies with the material type (fully graded, wet-screened, or core samples), loading regime (monotonic or cyclic), and strain rate.

At moderate preloading levels—typically up to 70–80% of the static tensile strength, within the elastic limit—several studies have observed an enhancement in dynamic tensile capacity [54,206,251,252]. The dynamic strength enhancement under static preloading can be a combined effect of the intrinsic strain rate enhancement and the weakening caused by static preload damage [113]. Weaker material generally exhibits a greater strain rate effect [16,155,156,160].

However, when the initial static load exceeds this threshold—particularly beyond 70% to 80% of the static strength—the trend reverses. Higher preloads tend to initiate and propagate microcracks at the aggregate–mortar interface and within the interfacial transition zone (ITZ), significantly weakening the tensile response [175,254]. Under dynamic or cyclic loading, these microcracks act as stress concentrators, triggering early failure and reducing both the strength and fracture energy. This is especially evident in splitting tensile tests, where concrete exhibits greater sensitivity to initial damage than in compressive scenarios [175].

Importantly, the type of loading (monotonic vs. cyclic) also mediates the role of the load history. While monotonic dynamic loads may show modest strength improvements with moderate preloading, cyclic loading—mimicking seismic conditions—often exacerbates damage accumulation, even at lower preload levels [47,206,250]. The energy dissipation and stress–strain hysteresis patterns under repeated loading cycles reveal that the preload accelerates fatigue damage and strain localization, particularly in large specimens or mass concrete [54,253].

Further complexity arises from the specimen scale and strain rate. Larger specimens, more representative of real dam concrete, show lower strength gains from preloading and are more susceptible to the amplification of the size effect when initial damage is present [175,249]. At higher strain rates, however, the adverse effects of preloading may be partially mitigated due to reduced deformation times, although this remains material- and structure-dependent [47,206].

Compressive preloading reduces concrete’s dynamic tensile strength by weakening internal microstructures, with the severity depending on the preload magnitude and repetition. Single compressive loads reduce the tensile strength by 10–25%, while repeated loading causes up to 60% loss [255]. The DIF at a strain rate of $10^{-2}$ s^−1^ can drop from 1.75 (no preload) to 1.24 after 30 cycles at 57% of the static compressive strain, confirming that the preload history significantly blunts rate-induced strength gains [209,256]. However, tension–compression preloading cycles can increase the DIF by 10–20% [257].

At high strain rates, prestatic loading significantly influences the dynamic tensile strength of concrete at high strain rates. When the preload is ≤50% of the static tensile strength, the dynamic strength remains stable across increasing strain rates due to minimal internal damage and effective microcrack resistance by aggregates. However, when the preload exceeds 75%, the dynamic tensile strength decreases notably, especially at higher strain rates [258].

To better understand and explain this phenomenon, researchers have explored numerically the effects of initial loads [100,113,174,186]. Wu and Zhang [186] explain that, as the strain energy in the elastic range produced by static preloading is prestored in the beam, such energy will instantly be released and transformed into kinetic and frictional energies at the instant of dynamic failure, thus resulting in a significant increase in total consumed energy and dynamic strength, leading to the failure of the beam.

### 7.2. Cyclic Loading

Experimental investigations into the cyclic tensile behavior of concrete reveal that, under certain conditions, the tensile strength under cyclic loads can be amplified compared to monotonic static loading. The parameters analyzed together with the cyclic load are the static preload, loading frequency, material characteristics, and their interactions. Flexural tests [251] demonstrated that three-graded dam concrete exhibited a progressive increase in the DIF (1.17 to 1.31) as the initial static preload rose from 0% to 80% of the static strength, with optimal performance at an 80% preload. Similarly, Chen et al. [54] observed that fully graded dam concrete achieved a plateau in the DIF (1.35) at a 40% preload under 5 Hz cyclic loading, indicating no further enhancement beyond this threshold. Excessive preloading, however, will degrade the DIF (1.29 to 1.24) when the static preload is higher than 80%, indicating a threshold [252].

Direct tension tests have highlighted the role of the load frequency: Wu et al. [259] reported higher DIF values at 5 Hz (1.19) compared to 1 Hz (1.04) under a 50% preload, while Lin et al. [250] noted DIF improvements (1.13 to 1.30) with increasing frequency (0.5–30 Hz) at a 72% preload, although amplitude increments had a minimal impact. Notably, excessive preloading (90%) reduced the DIF due to fatigue damage, similar to the findings of Zhou et al. [250,252]. The loading type also significantly influences the results: Darbar et al. [260] found that ramped-amplitude cyclic loading enhanced the DIF (1.30), whereas a constant amplitude reduced it (0.6–0.8), highlighting the detrimental effects of fatigue.

**Figure 6:**
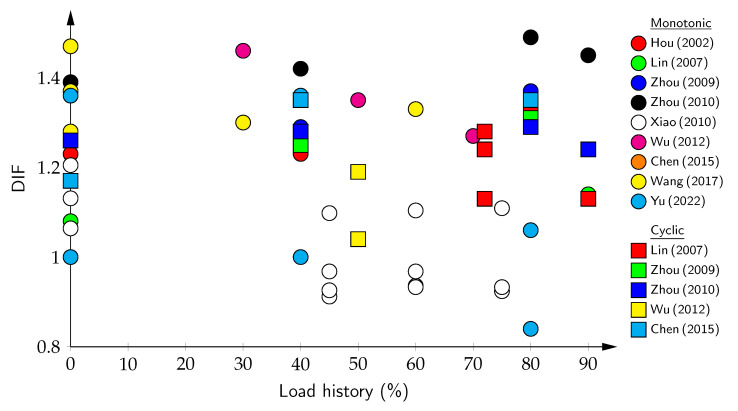
DIF as a function of preload level for monotonic and cyclic tension tests [47,54,175,206,249,250,251,252,253,259].

Compared to monotonic loading, cyclic loading generally exhibits slightly lower DIFs at equivalent preload levels (cyclic: 1.13–1.35 vs. monotonic: 1.23–1.49 at 40–80% preload) [54,251,252]. Figure 6 and Table 4 systematically compile experimental data revealing quantitative patterns in load history effects. For monotonic loading, the baseline DIF (0% preload) ranges from 1.00 to 1.47 with a mean of 1.24, increasing to 1.26 at 30–50% preloading and 1.30 at 60–80% preloading—representing progressive enhancements of 8% and 20%, respectively. However, significant scatter exists: at 60–80% preloading, individual studies report DIF values spanning 0.84 to 1.49, reflecting variations in the concrete type, strain rate (10^−5^ to 10^−2^ s^−1^), and material quality. However, Xiao [206] and Yu [175] reported reduced DIFs at high preloading (0.84–1.10), contrasting other studies showing enhancements (1.27–1.49), which opens up a discussion of this hypothesis.

## 8. Large-Aggregate Concrete

The dynamic properties of mass concrete are very important for the analysis and review of the seismic safety of concrete dams against strong earthquakes [261,262,263,264,265,266,267,268,269,270,271,272,273,274,275,276,277]. Furthermore, the characterization of the dynamic tensile strength is a key step in dynamic finite element analyses of the behavior of dams during earthquakes [278,279,280,281,282,283]. The dynamic tensile behavior of concrete shows clear rate sensitivity across the seismic strain rate range ($10^{-4}$ to $10^{-2}$ s^−1^). For large-aggregate concrete, defined as concrete with a nominal maximum size aggregate (NMSA) ≥40 mm, DIF values generally range between 1.1 and 1.8 depending on the strain rate range, concrete grading, aggregate size, moisture content, and load history, as evidenced by comprehensive research across dam cores and fully graded, three-graded, wet-sieved, or wet-screened and mass concrete [2,47,54,249,252,253,257,284,285].

Concrete grading plays a significant role in determining the strain rate sensitivity of dam concrete. Fully graded (or three-graded) dam concrete with large aggregates of up to 150 mm generally exhibits higher DIF values compared to wet-sieved (wet-screened or two-graded) concrete, where larger aggregates (greater than 40 mm) are removed. Wet-sieved concrete generally exhibits higher absolute static and dynamic tensile strength than fully graded concrete due to its reduced internal heterogeneity and improved matrix continuity [249,284,286,287,288]. However, the DIF is higher in fully graded concrete (1.6) compared to wet-sieved concrete (1.4), despite its lower baseline strength [249,284,286,287,288,289]. This indicates that, under dynamic loading, fully graded concrete benefits more from strain rate effects, which could be attributed to more extensive energy absorption mechanisms during aggregate fracture. Shen et al. [288] further emphasized that the growth rate of the DIF in dam concrete is significantly higher than that in ordinary concrete within the seismic strain rate range. This is a crucial insight for seismic safety assessment, where the relative toughness enhancement, rather than the absolute strength alone, often governs damage thresholds [288].

Concrete extracted from existing dams reveals how aging, microcracking, and real-world environmental exposure affect DIFs. Zhao [126] reported DIFs of 1.32 for cores from the Xiluodu Dam at $10^{-4}$ s^−1^, while Wang [253] observed DIFs up to 1.47 for cores from the Shapai Dam at $10^{-3}$ s^−1^. These values are consistent with historical data from Harris [285], who found DIFs averaging 1.44 and peaking at 1.73. These elevated DIFs support the conclusions of Raphael [2], who found that dynamic loading increased the tensile strength in dam cores by 50%. However, core-based data also show wide variability due to factors like aging, heterogeneity, and alignment with the aggregate orientation [2,285].

Fully graded dam concrete demonstrates strong strain rate sensitivity, with the DIFs increasing significantly with the strain rate. From flexural tests, DIFs were reported from 1.26 up to 1.8 [54,249,290]. Similarly, from uniaxial direct tension tests, DIFs have been reported to be as low as 1.1 and as high as 1.75 [47,256,284,286,287]. Cyclic loading reduces the dynamic enhancement, presenting DIFs of 1.17–1.39 [54,289]. Fully graded concrete can also be found in the literature as three-graded dam concrete [251,287,291]. In seismic strain rate ranges, the DIF for flexural tensile strength was observed to range from 1.2 to 1.6 [251,287,291]. It can be observed that the results are similar to those obtained from dam cores.

Concrete with large aggregates—common in mass concrete applications—exhibits distinct mechanical behavior under dynamic tension. Wu et al. [254] found DIFs ranging from 1.10 to 1.45 across test methods at strain rates of $10^{-3}$ to $10^{-2}$ s^−1^. In split tension tests, the results were similar to the DIFs found by Min et al. [53]. Corroborating the results of Wu et al. [254], from direct tension tests, DIFs can be found as low as 1.3 [292,293] and as high as 1.6 [176,294]. Saucier [257] provides lower bounds: 1.0–1.3 depending on moisture and precycling.

The interplay of large aggregates and moisture content markedly enhances the dynamic tensile behavior of concrete. Wu [47] noted an increase from a DIF of 1.08 (oven-dried) to 1.32 (fully saturated) at $10^{-3}$ s^−1^. Supporting this, both Harris et al. [285] and Saucier [257] observed 20–30% higher DIFs in saturated core specimens across various strain rates, confirming the critical role of free water in improving seismic resistance [47,257,285].

Studies have found that the loading history alters the dynamic response of large-aggregate concrete significantly. Initial static loads of up to 40–80% of the static strength generally enhance the DIF, as shown by Hou et al. [249] and Zhou [251], who reported DIFs rising from 1.20 to 1.50 at $10^{-3}$ s^−1^, although no increase was observed by Wang et al. [253]. Chen et al. [54] showed that cyclic loads reduced the DIF compared to monotonic loading, even under identical preloads. Zhou et al. [252] confirmed this: monotonic DIFs reached 1.49 (80% preload), while those for cyclic cases ranged only within 1.24–1.29. Bruhwiler [256] demonstrated that cyclic preloading—i.e., compressive—could reduce the DIF significantly (e.g., down to 1.07). Saucier [257] further validated these effects, showing that cyclic preloading in tension also introduces microstructural damage, suppressing strain rate sensitivity.

**Figure 7:**
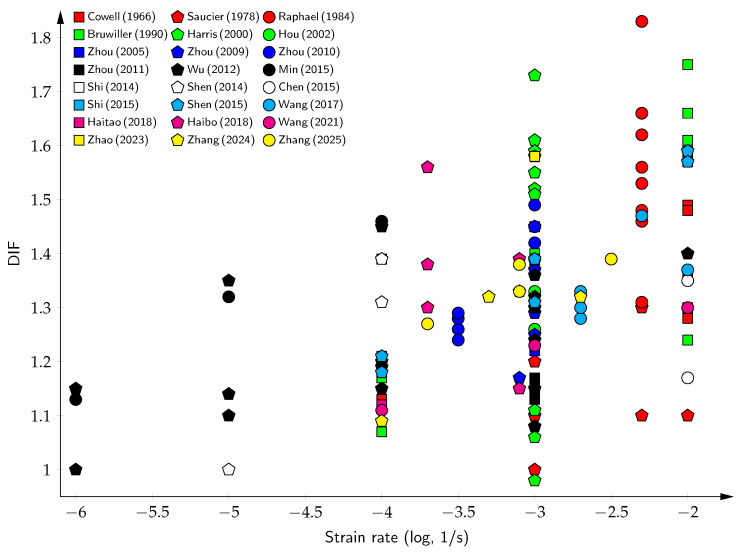
Results from experimental tension tests on large-aggregate concrete [2,16,47,53,54,176,249,251,252,253,254,256,257,284,285,286,287,288,289,290,291,292,293,294,295].

The available experimental results for large-aggregate concrete are presented in Table 5. For strain rates in the range of $10^{-3}$ to $10^{-2}$ s^−1^, which correspond to dam vibrations at frequencies of 5–10 Hz, the average DIF is approximately 1.6. This value can be either enhanced or diminished depending on factors such as the moisture conditions, load history, and concrete grading. As shown in Figure 7, despite the sparse data distribution, a discernible trend emerges. With increasing strain rates, the DIF can range from 1.1 to 1.8.

## 9. Concrete Quality

Another factor influencing the dynamic tensile strength of concrete is the material quality. Research indicates that the effect of the strain rate diminishes as the concrete quality improves [16,155,156,160]. High-performance concretes (HPCs), for example, tend to show reduced sensitivity to the loading rate when compared to ordinary concretes [182]. This behavior has been attributed to improved microstructural characteristics, such as higher quasi-static compressive strength, reduced porosity, and lower water/cement ratios. The water-to-cement ratio (*w/c*) directly affects this relationship: concretes with higher *w/c* ratios exhibit greater strain rate sensitivity due to increased free water content and capillary porosity, whereas low *w/c* mixes—typical of HPC or UHPC—display a weaker rate dependence [181,182]. Beyond its effect on porosity, the *w/c* ratio also governs key mechanical parameters, such as the modulus of elasticity and Poisson’s ratio. Increasing the *w/c* ratio reduces the degree of hydration and substantially decreases the fracture toughness, thereby amplifying the strain rate sensitivity [179,296].

Aggregate characteristics, including the maximum size and shape, also play a role in rate sensitivity. Some studies suggest that larger aggregates may lead to increased rate sensitivity due to scale effects, differential crack development, or cement paste behavior around aggregate inclusions. Nevertheless, the relationship between the aggregate size and strain rate effect remains complex and is still a topic of ongoing investigation [297,298]. Beyond the aggregate size, the degree of aggregate packing and compaction also influences rate effects—well-compacted mixes tend to show lower sensitivity to the strain rate than loosely packed ones, reflecting the role of the porosity and microcrack density [254,299].

**Figure 8:**
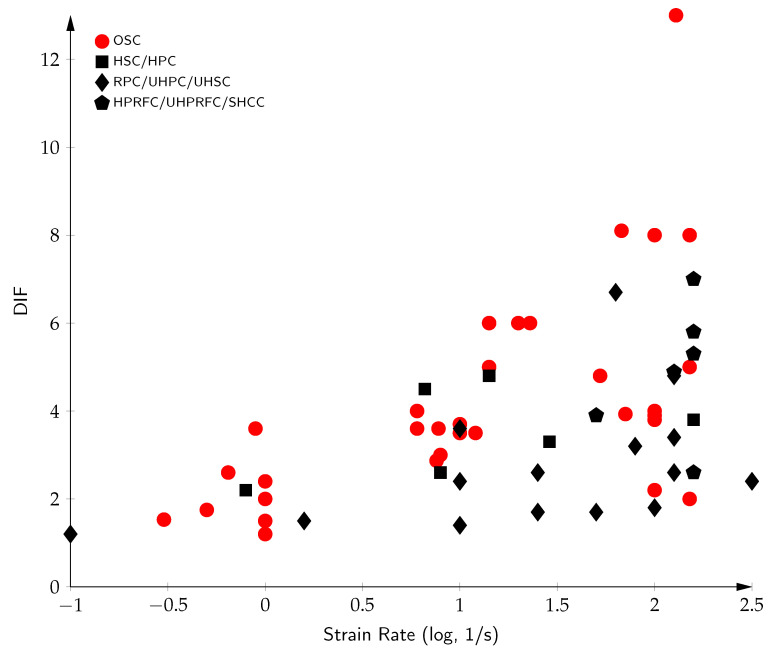
DIF as a function of concrete quality at high strain rates.

A consistent observation across studies is the inverse relationship between the concrete quality and strain rate sensitivity. Concretes with lower compressive strength tend to exhibit greater increases in tensile strength under dynamic loading than stronger, more brittle concretes. This implies that the DIF is more pronounced in lower-grade concretes. As such, while dynamic loading conditions elevate the tensile strength across all types of concrete, the magnitude of this effect is reduced in high-performance materials [182,297,298]. This trend is illustrated in Figure 8, which compares the DIF across concretes of different quality levels at high strain rates, from the data available in the literature [5,6,18,19,22,24,28,29,31,37,38,39,44,45,46,49,50,51,52,56,57,58,59,60,61,63,65,66,67,68,69,73,74,78,79,163,165,171,179,300,301,302,303,304,305,306,307]. Ordinary-strength concretes (OSCs) exhibit the largest DIF values at high strain rates (DIFs of 1.2–6 at 0.1–150 s^−1^), while high-performance and ultra-high-performance concretes display significantly reduced rate sensitivity (DIFs of 2.0–4.9 at 21–420 ss^−1^), confirming the inverse correlation between concrete quality and the strain rate effect.

Supplementary cementitious materials (SCMs) such as silica fume, fly ash, and ground granulated blast furnace slag (GGBS) further modulate this behavior. Silica fume and GGBS tend to increase the dynamic strength by refining the matrix and improving interfacial bonding, whereas fly ash may reduce the DIF at lower strain rates by mitigating brittleness [140]. Cement content and chemical admixtures can further influence the dynamic response. Concretes with higher cement content often exhibit lower uniaxial impact tensile strength, likely due to the reduced bond strength between aggregate particles and the cement matrix [179]. However, high-strength cement-based materials such as reactive powder concrete (RPC) show no apparent differences in strain rate sensitivity compared to normal concretes, indicating that the ultimate strength alone does not govern rate effects [170].

A mechanistic explanation for this trend has been proposed based on internal damage evolution. In lower-quality concrete, the heterogeneous microstructure allows dynamic cracks to activate a network of weak zones, generating widespread microcracking that delays final fracture and contributes to enhanced strength. Conversely, in higher-quality concretes—such as ultra-high-performance concrete (UHPC) and ultra-high-performance geopolymer concrete (UHPGC)—homogeneity and reduced porosity constrain the development of such damage. Under high strain rates, cracks are forced to traverse more resistant paths, but the number of microcracks remains limited. Consequently, the internal damage profile does not differ substantially between quasi-static and dynamic loading conditions, leading to reduced rate sensitivity [80]. At the microstructural level, the quantity of hydrated calcium silicate (CSH) crystals also influences the rate-dependent response. Higher CSH content enhances the absolute increase in tensile strength per logarithmic unit of the loading rate, serving as a key indicator of improved resistance under dynamic loading [163,299].

In addition to porosity and strength effects, moisture and water distribution within the cement matrix play a critical role. At moderate strain rates, free water in nanopores generates viscous resistance to crack propagation (Stefan effect), producing higher apparent strain rate sensitivity in moist or saturated concretes compared to dry specimens [41,163,296].

In fiber-reinforced concretes like ultra-high-toughness cementitious composites (UHTCCs), fiber bridging slows down crack propagation, which reduces the crack opening velocity and consequently diminishes the Stefan effect. The altered crack path caused by fibers brings free water closer to the crack tip, reducing the capillary resistance to crack growth. While viscous effects contribute to the dynamic strength of the concrete matrix, their influence weakens in fiber-bridged zones, where extensive microcracking around fiber anchorage points limits the action of these mechanisms. Thus, fiber reinforcement modifies both the fracture process and the role of viscous phenomena, particularly after cracking [188,296]. The influence of the fiber type also varies: polypropylene fibers generally enhance ductility and slightly reduce the strain rate sensitivity, whereas small steel fiber content (below 2%) shows a minimal impact on the DIF [170].

Under moderate strain rates, UHPC exhibits typical DIFs ranging from 1 to 1.5 within the range of $10^{-4}$ to $10^{-2}$ s^−1^ [153], which is comparable to the values observed for ordinary concrete. At high strain rates ($10^{2}$ s^−1^), the DIF for UHPC typically reaches values between 4 and 6 [153,188], whereas, for ordinary concrete under dry conditions, the DIF is generally in the range of 6 to 8 [5,6,22].

## 10. High Strain Rates

The tensile strength of concrete exhibits significant sensitivity at high strain rates from impacts caused by loads such as blasts and explosions. High strain rates—typically in the range of $10^{1}$ s^−1^ to $10^{2}$ s^−1^ and above—can cause pronounced increases in strength [308], with some experimental studies reaching strain rates up to $10^{3}$ s^−1^ [65,81]. At these rates, the tensile strength increases markedly, and the DIF can reach values close to 13 [5,152,170].

Several physical mechanisms have been proposed to account for this substantial rate-dependent increase in tensile strength, particularly above a critical threshold estimated around 1 s^−1^. One such mechanism is microcracking inertia, whereby the inertia of the material under high strain rates resists the localization and rapid propagation of cracks [5,41,245]. Microcrack shielding is another explanation, suggesting that rapidly forming cracks interfere with one another, delaying unstable crack growth [5,41]. At even higher strain rates, aggregate cleavage may occur due to insufficient time for cracks to divert around aggregates, leading to their direct splitting [5,35,41]. Furthermore, the limited crack propagation velocity necessitates higher stress levels to achieve failure, contributing to the observed strength gains [184].

Additional factors include pore water pressure effects, wherein saturation under rapid loading conditions elevates the pore pressure, influencing the apparent strength. However, in the transition range between 1 s^−1^ and 10 s^−1^, this effect becomes less dominant as the intrinsic material rate sensitivity increases [5,184,309]. Finally, the concept of the multi-activation of fracture processes posits that the material’s response becomes more uniform under dynamic conditions, reducing the influence of individual flaws and promoting the simultaneous formation of multiple cracks, which collectively enhance load resistance [5,178,179,309].

Beyond research on the strain rate sensitivity of plain concrete, the dynamic response of high-performance concrete has been invariably evaluated at high strain rates. Table 6 compiles tensile test data obtained at high strain rates and reveals clear differences among concrete types. Ordinary concrete (OSC) exhibits a pronounced strain rate sensitivity, with dynamic increase factors (DIFs) generally ranging from 1.5 to 6 over strain rates between $10^{-1}$ s^−1^ and $10^{2}$ s^−1^. This increase follows an approximately logarithmic trend and is strongly influenced by the moisture conditions: wet concretes consistently show higher DIFs than dry ones because of pore water pressurization and microcrack closure effects. In contrast, high-strength concrete (HSC) presents a more moderate response, with DIFs typically between 2 and 4.8 at comparable strain rates. The dense microstructure and strong aggregate–paste interface of HSC limit crack growth and reduce the rate sensitivity, although water saturation still enhances the apparent strength.

High- and ultra-high-performance concretes (HPC, UHPC, and their fiber-reinforced variants) demonstrate comparatively mild strain rate dependences, with most DIFs between 1.2 and 3.6. Their refined pore structures and high intrinsic tensile strength mitigate inertial effects, while the inclusion of fibers can locally increase the DIF to values around 5–6 owing to rate-dependent fiber bridging and pullout mechanisms. In contrast, dam or mass concrete, characterized by large aggregate sizes and low cement content, generally shows lower DIFs of about 2–3, even under similar loading rates. The coarse grading and heterogeneity of such mixes promote early crack localization and reduce the apparent strengthening effect.

Overall, the results indicate that the DIF increases with the strain rate up to approximately $10^{2}$ s^−1^, beyond which it tends to stabilize. The magnitude of this increase is inversely related to the material quality: concretes with higher strength and compactness exhibit smaller relative DIF slopes. Moisture and fiber reinforcement emerge as consistent amplifying factors—the former through transient pore pressure and the latter through enhanced energy absorption during dynamic crack propagation.

## 11. Numerical Simulation

The understanding of concrete’s dynamic tensile behavior has been greatly advanced through numerical modeling, which captures the complex interplay between mesostructural heterogeneity, strain rate sensitivity, crack evolution, and inertial effects. In recent decades, researchers have utilized diverse modeling approaches including the finite element method (FEM), the discrete element method (DEM), peridynamics (PD), and coupled continuum–discrete frameworks to simulate dynamic tension scenarios such as split Hopkinson pressure bar (SHPB) tests, direct tension, and spalling.

The FEM is widely employed in simulating dynamic tensile fracture using continuum-based constitutive models. Among the most common are the concrete damaged plasticity (CDP) model [35,107,310], Holmquist–Johnson–Cook (HJC) [133,135,311], Karagozian and Case (K&C) [78,130], and, less frequently, the Drucker–Prager and Riedel–Hiermaier–Thoma (RHT) models [312,313]. The Kamran and Iqbal (K&I) model has also been introduced recently for strain rate-sensitive concrete modeling [314].

These constitutive laws are embedded in FEM simulations, representing concrete as a three-phase composite—mortar, aggregate, and the interfacial transition zone (ITZ)—with strain rate effects introduced either via empirical DIF formulations or directly through rate-sensitive damage or viscoplasticity models [108,115,128]. Cohesive zone models (CZMs), including zero-thickness cohesive elements with traction–separation laws, have proven effective in simulating crack nucleation and propagation across aggregate–mortar interfaces [83,134,138].

Viscous regularization frameworks, such as Perzyna-type viscoplastic damage models, mitigate mesh dependency and capture the rate-dependent softening process under dynamic loading [84]. More recently, thermal–mechanical simulations including ice expansion-induced prestress have been applied to simulate the dynamic fracture of cold-region concrete [128].

The discrete element method (DEM) has proven powerful in simulating stochastic fracture, crack branching, and fragmentation. Variants include bonded particle models (BPMs) and rigid body spring models (RBSMs) [91,92]. In BPMs, aggregates are treated as rigid particles with bonds representing cohesive strength; breakage criteria are often rate-sensitive and based on Weibull distributions [30,125]. The DEM excels in capturing mesofracture processes—such as interface crack initiation, tensile spalling, and projectile impact fracture—especially under high strain rates [3,30,135]. However, its high computational cost and calibration demands often limit its use to small-scale or idealized scenarios. Some researchers have used the DEM to simulate fiber bridging effects [126] or to model concrete with extreme heterogeneity, such as coral aggregate seawater shotcrete, where micromechanical properties were derived from nanoindentation [136].

Peridynamics, a nonlocal meshless method, bypasses classical crack tip singularity issues and allows arbitrary crack path simulation. PD has been used to simulate fracture in saturated concrete, incorporating pore pressure and capillary cohesion [124]. While highly accurate in crack pattern prediction, PD’s computational intensity limits its scalability.

Coupled continuum–discrete frameworks—such as FDM-DEM models—bridge the gap between wave propagation and fracture. For example, Peng et al. combined the finite difference method (FDM) for steel bar dynamics with the discrete element method (DEM) for concrete fracture in coral aggregate shotcrete [136]. Zhou et al. coupled the FDM and DEM to model Brazilian discs with interface cracks, replicating both wave transmission and acoustic emissions [137]. A novel direction involves discrete element method and computational fluid dynamics (DEM-CFD) coupling, simulating fluid migration during dynamic fracture. Krzaczek et al. [129] showed how water trapped in pores under fast tensile loading contributes to the Stefan effect, transiently enhancing the tensile strength through viscous confinement.

A recurring finding across nearly all validated models is that concrete exhibits intrinsic strain rate sensitivity in tension, beyond what can be explained by inertial effects alone. When material models lack proper rate enhancement—such as DIFs or viscoplastic hardening—they tend to underpredict the dynamic tensile strength at moderate strain rates ($10^{0}$ to $10^{2}$ s^−1^) [35,84,120]. Rate-dependent formulations within CDP, HJC, or cohesive laws offer much closer alignment with SHPB or spall test data [78,128,135].

At high strain rates (>1 s^−1^), inertial effects increasingly influence strength and fracture patterns. These include stress wave reflections, delayed strain localization, and stress confinement, all of which raise the apparent dynamic strength even in the absence of an enhanced material response [3,83,93,137]. Some researchers isolate this inertial contribution using techniques like the equivalent momentum scheme (EMS) [3].

Moisture content exerts a dual effect. At low saturation, water weakens the matrix and ITZ, reducing the tensile strength [118,121]. However, at high saturation levels, especially under dynamic loading, water generates beneficial effects, including pore pressure stiffening, viscous resistance, and capillary adhesion—collectively enhancing the dynamic strength [124,129,238]. Nevertheless, these saturated concretes may become more brittle and less energy-absorptive, exhibiting sharper post-peak behavior [77,96].

The crack path and morphology are tightly linked to the mesostructure and strain rate. At low rates, cracks follow the ITZ or mortar phase, but, at higher strain rates, fractures propagate through aggregates due to reduced crack development times and higher inertia [117,122]. Several multi-phase FEM and DEM models have confirmed that crack branching, fragmentation, and secondary fracture zones become prominent at elevated loading rates [125,136].

The specimen size and geometry also impact the dynamic tensile strength. While larger specimens show lower quasi-static strength, this size effect is diminished or reversed under dynamic conditions, primarily due to inertia-dominated stress redistribution and delayed failure [3,114,205]. Simulations matching SHPB setups have confirmed this via time-to-failure and energy absorption trends.

In conclusion, numerical simulation has enabled a deeper understanding of the interplay between the strain rate, mesostructure, moisture, and inertia in concrete’s dynamic tensile behavior. Ongoing research increasingly favors 3D, physically grounded, and multi-scale frameworks validated against high-speed testing and imaging. Future efforts should prioritize integrating hybrid models, experimental–numerical calibration, and machine learning-aided parameter optimization to increase the accuracy and scalability for critical applications like blast-resistant infrastructure and seismic protection.

The numerical approaches summarized in Table 7 present distinct trade-offs between accuracy, computational efficiency, and implementation complexity. The FEM with continuum constitutive models (CDP, HJC, K&C) remains the most practical choice for large-scale structural analyses, offering mature commercial software support and the efficient handling of wave propagation, although it struggles with arbitrary crack propagation and exhibits mesh dependency [107,130,133]. CZMs partially address crack simulation limitations but require predefined interface locations [83,134]. Mesoscale FEM approaches explicitly representing aggregates, mortar, and ITZs provide superior insights into fracture mechanisms and material heterogeneity effects but demand significantly greater computational resources and extensive material characterization [95,115,128]. The DEM naturally handles fragmentation, multiple cracking, and discontinuities without mesh distortion, making it ideal for spalling and high-rate fracture simulation [3,30], yet it suffers from extreme computational costs, challenging calibration procedures linking microparameters to macroproperties, and limited accuracy in elastic wave propagation [118,123]. PD offers theoretical elegance for crack branching and coalescence through nonlocal formulations that inherently accommodate discontinuities, and recent implementations have successfully incorporated pore pressure and fluid coupling for Stefan effect modeling [124,129], but its computational expense exceeds that of the DEM, and commercial software support remains limited. Coupled continuum–discrete frameworks (FDM-DEM, DEM-CFD) represent the frontier in capturing complex coupled phenomena—efficiently handling intact material responses while accurately simulating fracture and fluid migration—albeit requiring custom implementation and expert knowledge [129,136,137]. Practical selection depends on research objectives: the continuum FEM for structural-scale problems prioritizing efficiency, the mesoscale FEM or DEM when detailed fracture mechanisms are critical, and advanced coupled methods for fundamental research on multi-physics interactions. All approaches require careful experimental validation of not only strength predictions but also crack patterns and energy dissipation.

## 12. Dynamic Properties in Guidelines for Concrete Dams

Following research on the dynamic properties of concrete [2,256,315], guidelines for the design of concrete dams consider that concrete, when subjected to dynamic loading, may exhibit characteristics that differ from those under static loading. The strength and elastic properties are assumed to be linearly amplified by the DIF. Table 8 summarizes the DIF values provided in the guidelines for concrete dams.

The dynamic tensile strength presented by the United States Army Corps of Engineers (USACE) [265] is based on the modulus of rupture test for concrete used in dams and is modified according to the approach discussed by Raphael [2]. Tests performed at loading rates typical of earthquake excitations, conducted by Raphael [2], have shown that, on average, the tensile strength increases by about 50%. This value is widely adopted in various guidelines [266,267,268,269,270,272,276,277]. The DIF stated by the USACE [270] is based on the investigation of Bruhwiler [256].

In general, an increase of 30% in the dynamic compressive strength relative to the static value is recommended in the guidelines [265,267,271,275]. The increases proposed by Hydro-Quebec [268], the USBR [269], and the USACE [270] are based on recommendations from the National Research Council (NRC) [316], laboratory tests conducted by the United States Bureau of Reclamation (USBR), and Bruhwiler [256], respectively. No amplification is considered by the USBR [261,262,263,269], the Australian National Committee on Large Dams (ANCOLD) [277], or the International Commission on Large Dams (ICOLD) [272].

Earlier USBR guidelines [261,262,263], as well as the USBR [269], indicate that the instantaneous modulus of elasticity determined from concrete specimens at the time of initial loading should be used in analyses of dynamic effects. In the USBR [264] and USACE [265], this value is recommended to be increased by 20%. Currently, an increase of 25% in the modulus of elasticity is recommended by the Federal Energy Regulatory Commission (FERC) [267], Hydro-Quebec [268], the Swiss Office of Energy (OFEN) [275], the Swedish Energy Research Centre (SERC) [276], and ANCOLD [277].

A DIF of 1.25 is recommended by Hydro-Quebec (HQ) [268] for Poisson’s ratio, based on the value proposed by the USACE [265]. In contrast, the USACE [270] recommends a reduction in Poisson’s ratio, with a DIF of 0.7.

### Comparison with DIF Formulations

Across the strain rate range representative of seismic loading ($10^{-4}$ to $10^{-2}$ s^−1^), most empirical and semi-empirical formulations for the dynamic increase factor (DIF) of concrete tensile strength yield values between 1.2 and 1.5 at the upper bound of this range, which aligns closely with the constant DIF values recommended in major dam design guidelines (typically 1.0–1.7, most commonly 1.5, as detailed in Table 8).

**Figure 9:**
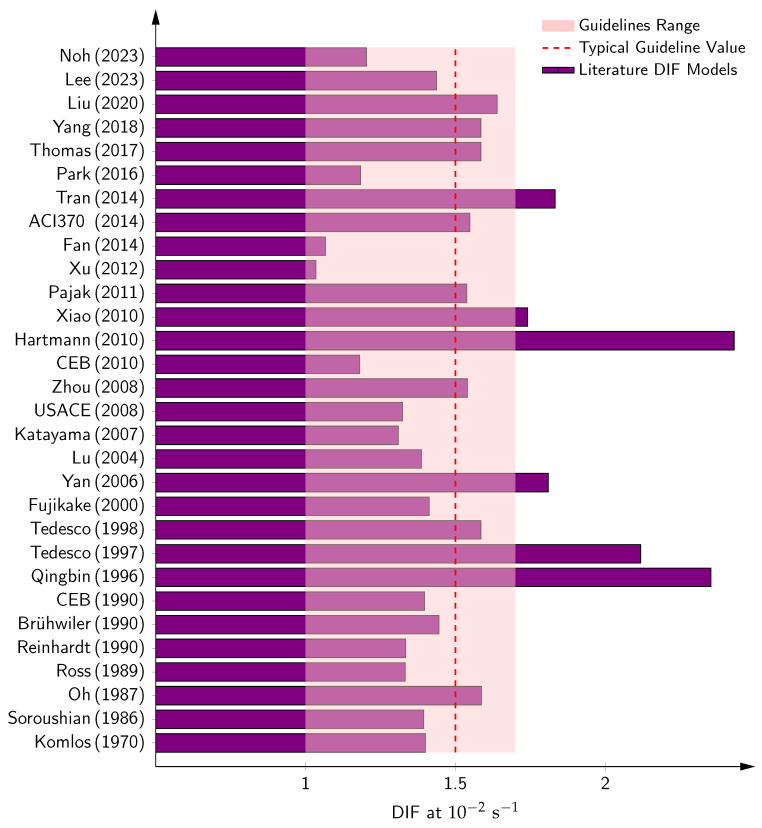
Comparison of DIFs from literature [23,24,36,66,145,153,155,156,170,187,200,203,204,205,206,207,208,209,210,212,213,214,216,217,218,219,220,221,222,223,225] vs. dam design guidelines [261,262,263,264,265,266,267,268,269,270,271,272,273,274,275,276,277].

Figure 9 provides a systematic comparison of the DIF values at strain rate $10^{-2}$ s^−1^ predicted using literature formulations (compiled in Table 2) against the recommended ranges in international dam design guidelines. The convergence of most models within or near the guideline range (1.0–1.7, indicated by the shaded region) validates the current engineering practice while highlighting that the typical guideline value of 1.5 (dashed line) represents a reasonable rather than conservative estimate.

## 13. Discussion

This comprehensive review has synthesized the current understanding of concrete’s dynamic tensile behavior across mechanisms, experimental methods, predictive formulations, and engineering applications. While substantial progress has been achieved, critical gaps remain for dam engineering practice. This section examines areas of consensus and contradiction, evaluates methodological limitations, and identifies priority research directions.

### 13.1. Mechanisms

The material’s inherent strain rate sensitivity arises from the combined effects of microcracking, viscosity, and structural inertia. A debate persists regarding the relative dominance of these mechanisms. Most studies agree that both contribute, with inertial effects becoming increasingly significant at higher strain rates, often reflected in the measured apparent strength. Some researchers emphasize the primacy of intrinsic material behavior, particularly in tension, while others highlight the growing influence of inertial forces under dynamic loading.

**Figure 10:**
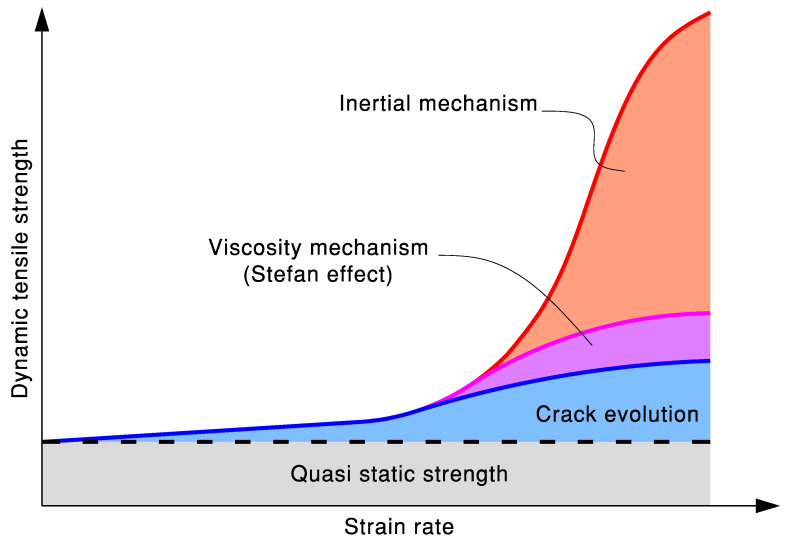
Schematic decomposition of dynamic tensile strength enhancement mechanisms.

From an extensive review of the literature, it can be observed that dynamic tensile strength enhancement fundamentally arises from mechanisms that delay crack propagation across different strain rate regimes. A schematic decomposition of these mechanisms is illustrated in Figure 10. In this framework, the baseline crack evolution exhibits moderate rate sensitivity, which is further amplified by viscous and inertial mechanisms. From seismic to moderate strain rates ($10^{-4}$ to 1 s^−1^), viscous mechanisms dominate: the Stefan effect generates additional resistance as free water trapped between crack surfaces physically retards the crack opening velocity, with the magnitude proportional to the porosity and moisture content. At high strain rates (>1 s^−1^), inertial mechanisms increasingly dominate, as the material’s resistance to rapid acceleration produces confinement effects. Future models must capture the transitions and interactions between these mechanisms to enable reliable predictions across the full strain rate spectrum.

### 13.2. Established Knowledge and Uncertainties

The field has achieved a consensus on several fundamental aspects. The positive strain rate sensitivity of concrete tensile strength is universally confirmed, with the DIF increasing monotonically across all rate regimes. For seismic loading ($10^{-4}$ to $10^{-2}$ s^−1^), the convergence of experimental data, empirical formulations, and guideline recommendations on DIF values of 1.2–1.8 provides confidence for engineering applications. The inverse relationship between concrete quality and rate sensitivity is consistently observed: ultra-high-performance concrete exhibits approximately 30% lower DIFs than ordinary concrete, attributed to reduced porosity limiting moisture effects and distributed microcracking. Most remarkably, moisture content dominates dynamic behavior through the Stefan effect, producing a 2–3× DIF amplification in saturated versus dry concrete at moderate-to-high rates (>1 s^−1^).

However, fundamental uncertainties exist. The separation of structural inertial effects from intrinsic material rate sensitivity remains contentious, with some researchers attributing most high-rate strength enhancements to inertia, while others demonstrate material effects even after correction. This disagreement stems from different experimental configurations emphasizing different mechanisms, a lack of standardized decomposition methods, and difficulty in validating numerical approaches. The practical consequence is uncertainty in applying DIF values from laboratory tests (which include inertia) to finite element models (which compute inertia separately). Evidence suggests that inertial contributions become significant above 1–10 s^−1^ depending on the specimen geometry, but quantification remains configuration-specific.

Load history effects show contradictory trends across studies, with some reporting DIF enhancements under moderate preloading, while others observe degradation at similar levels. These discrepancies may arise from differences in preloading protocols, but they also reflect complex interactions between damage accumulation, moisture redistribution, and material heterogeneity that are not consistently controlled or reported in experiments. The reversal of size effects under dynamic loading—from negative quasi-statically to positive dynamically—is well documented but poorly predicted, with transition strain rates varying by an order of magnitude across studies. This uncertainty complicates the scaling of laboratory results to massive structures.

### 13.3. Knowledge Gaps

Gaps can be identified in the literature. Material-specific knowledge is severely lacking for dam-relevant concretes. Large-aggregate concrete (NMSA ≥ 40 mm) appears in few studies at seismic rates. The role of free water could be further studied across different saturation levels, including comparisons between air-dried and oven-dried specimens. The impact of preloading remains ambiguous, with ongoing debate about whether it improves or degrades strain rate sensitivity. Additionally, the contribution of the aggregate size and its interaction with the overall concrete quality warrant deeper investigation. The effect of inertial forces at high strain rates should also be explored more rigorously, both experimentally and through numerical simulations.

### 13.4. Engineering Practice and Research Priorities

A comparison of guidelines with the literature has been made in this review. The guideline adoption of DIF ≈ 1.5 for seismic tensile strength is reasonable, supported by the convergence of DIF formulations (mean = 1.50) with guideline ranges (typically 1.0–1.7). However, refinements would improve the accuracy: specifying whether the DIF applies to dry, partially saturated, or fully saturated concrete; accounting for the load history; providing material-specific DIF ranges based on concrete quality and saturation; and clarifying the conditions under which local strain rate concentrations justify a rate-dependent rather than constant DIF’s application.

Research priorities emerge clearly from the identified gaps, which include developing standardized testing protocols for saturation, preloading, large-aggregate testing, and inertia correction; conducting experimental studies on large-aggregate concrete at seismic rates with comprehensive documentation; and quantifying saturation level (0–100%) and preloading level (0–80%) effects for implementation in DIF formulations.

At a secondary level, additional gaps can be covered too, which include implementing Stefan effect representation through coupled formulations; validating numerical inertia decomposition approaches against systematic experiments; developing geometry-dependent correction factors for high-rate data; establishing size effect scaling laws through the testing of multiple specimen sizes; and conducting factorial experiments quantifying damage–moisture rate interactions.

For long-term dam safety assessment, the following aspects may be included: large-scale validation testing of dam sections; performance-based design frameworks incorporating rate-dependent fracture energy and deformation limits; the integration of performance data from instrumented dams experiencing earthquakes; and updated design guidelines reflecting these advances.

## 14. Conclusions

This paper presents a comprehensive review of the dynamic tensile strength of concrete, emphasizing its critical role in the safety and performance of massive structures such as dams under extreme dynamic events like earthquakes. At the core of the discussion lies the concept of the dynamic increase factor (DIF), which quantifies the enhancement in concrete’s tensile strength under dynamic loading conditions compared to its static performance. DIF values typically range from 1.1 to over 12, depending on factors such as the strain rate, load history, concrete composition, moisture content, and test setup. Key findings include the following:

1. DIFs of 1.2–1.8 are well established for seismic strain rates ($10^{-4}$ to $10^{-2}$ s^−1^), with a consensus between experimental data, empirical formulations, and international design guidelines.
2. Moisture content significantly influences the DIF through the Stefan effect. At seismic strain rates ($10^{-4}$ to $10^{-2}$ s^−1^), saturated concrete exhibits DIF values of 20–50% higher than those of dry concrete, with the effect becoming more pronounced at higher strain rates within this range. At rates >1 s^−1^, moisture amplification increases to 2–3×, yet moisture effects are rarely addressed in current design codes.
3. Moderate preloading (up to 70–80% of the static tensile strength) within the elastic limit can enhance the dynamic tensile strength by 10–25%, while excessive preloading (>80%) causes degradation.
4. Ultra-high-performance concrete exhibits ∼30% lower DIFs than ordinary concrete at equivalent strain rates, confirming an inverse quality–rate sensitivity relationship.
5. Inertial effects contribute 20–50% of the apparent strength enhancement at high rates (>10 s^−1^) but are negligible for seismic analysis (<1 s^−1^).
6. Strain rate effects on large-aggregate dam concrete (NMSA ≥ 40 mm) remain critically understudied, with only 15 investigations conducted at seismic rates, compared to 100+ studies on conventional concrete.
7. Cyclic loading produces slightly lower DIFs (5–10% reduction) compared to monotonic loading at equivalent preload levels due to fatigue damage accumulation, with frequency effects—higher frequencies leading to higher DIFs.
8. The current design guidelines are validated by comparison with research-based formulations but should be refined to specify moisture and load history conditions.
9. No existing constitutive model simultaneously captures the rate sensitivity, moisture effects, and load history in a unified framework.
10. For engineering practice, a DIF = 1.5 is recommended for the seismic analysis of dams, with a 20–30% increase for saturated conditions and a 10–15% increase accounting for operating hydrostatic loads.

This review uniquely provides the first comprehensive synthesis explicitly focused on large-aggregate dam concrete at seismic strain rates ($10^{-4}$ to $10^{-2}$ s^−1^); the systematic integration of moisture, load history, and material quality effects, which are rarely addressed together; a critical evaluation of international dam design guidelines against state-of-the-art research; a quantitative comparison of DIF formulations with guidelines and the presentation of an experimental database; and a prioritized research roadmap and practical impacts.

## Figures and Tables

**Table 1:** Summary of techniques applied in dynamic testing of concrete.

Classification	Load Type	Technique	Strain Rate (1/s)	Method Description
Static to quasi-static	Earthquake Construction Traffic Vehicle impact	Servo-hydraulic	$10^{-7}$ to $10^{1}$	Applies loads to a specimen using a hydraulic actuator
Intermediate strain rates	Traffic Vehicle impact Crashworthiness Collisions Construction Aircraft landing	Dropkinson	$10^{0}$ to $10^{2}$	Dropkinson test combines a drop tower and a Hopkinson bar
		Drop Hammer Drop Tower	$10^{0}$ to $10^{2}$	Drops a heavyweight hammer or a mass to impact a clamped specimen
		High-Speed Servo-Hydraulic	Up to $10^{3}$	Designed to cope with the dynamics of intermediate strain rate testing
		Hybrid Servo-Hydraulic Hopkinson Bar	Up to $10^{3}$	Combines a servo-hydraulic machine and the split Hopkinson bar technique
Intermediate to high strain rates	Blast Explosion Vehicle impact Hard impact	Split Hopkinson Pressure Bar (SHPB)	$10^{1}$ to $10^{5}$	Involves two bars with a specimen placed between them A striker bar generates stress waves that propagate through the specimen
		Modified Hopkinson Bar	$10^{1}$ to $10^{3}$	Variation of the SHPB designed for tensile testing
High strain rates	Induced shock Hard impact Blast Explosion	Expanding Ring	$10^{2}$ to $10^{4}$	Impact load is applied internally to thin ring specimens by the sudden radial acceleration of the driving ring due to the detonation of an explosive charge or electromagnetic loading
		Shock Tube	$10^{2}$ to $10^{5}$	A specimen placed at the end of a tube is exposed to a shock wave impact
		Rotating Wheel or Flywheel	$10^{3}$ to $10^{4}$	Uses the kinetic energy from a rotating flywheel to fracture the specimen
Very high strain rates	Blast Explosion Induced shock	Taylor Test or Rod Impact	$10^{4}$ to $10^{5}$	Launches a cylindrical specimen that impacts a rigid target
		Explosion	$10^{2}$ to $10^{5}$	Specimen is subjected to blast loading generated by explosives
Very high to ultra-high strain rates	Bullet impact Explosion	Pressure Shear Plate Impact	$10^{4}$ to $10^{6}$	An inclined flyer plate impacts the inclined target plate specimen
		Plate Impact Flyer Plate Test	$10^{4}$ to $10^{8}$	Impacts a flat plate on a target sample in a planar manner

**Table 3:** DIF in tension for dry and wet concrete.

Reference	Strain Rate Range (s^−1^)	DIF (Dry)	DIF (Wet)
Reinhardt [24]	$10^{-1}$–$10^{0}$	0.9–1.1	1.4–3.1
Rossi [173]	$10^{-5}$–$10^{1}$	1.0–1.7	1.0–4.7
Ross et al. [28]	$10^{-7}$–$10^{1}$	0.9–3.0	0.9–4.0
Toutlemonde and Rossi [244]	$10^{-6}$–$10^{0}$	1.0–1.4	1.1–2.4
Cadoni et al. [29]	$10^{-6}$–$10^{1}$	1.0–1.7	1.0–3.1
Brara and Klepaczko [5]	$10^{-6}$–$10^{2}$	1.0–8.7	1.0–12.6
Wang et al. [236]	$10^{-2}$–$10^{1}$	1.7–2.1	1.9–2.9
Erzar and Forquin [6]	$10^{1}$–$10^{2}$	3.5–6.1	4.5–8.3
Wu et al. [47]	$10^{-6}$–$10^{-3}$	0.9–1.4	1.3–1.4
Vegt and Weerheijm [190]	$10^{-6}$–$10^{2}$	1.0–3.9	1.0–8.4
Wang et al. [248]	$10^{-5}$–$10^{-2}$	1.0–1.6	1.0–1.8
Cui et al. [78]	$10^{-1}$–$10^{1}$	1.1–5.7	1.6–6.0
Xie et al. [238]	$10^{1}$–$10^{2}$	3.7–4.8	4.5–6.3
Liu et al. [82]	$10^{-5}$–$10^{-1}$	1.0–1.6	1.0–2.5

**Table 4:** Summary of DIF values for concrete under various preload levels and loading conditions.

Study	Strain Rate, 1/s/ Frequency, Hz	DIF as Function of Preload Level			
		No Preload	30–50% Preload	60–80% Preload	90% Preload
Monotonic					
Hou [249]	$10^{-3}$	1.23–1.26	1.23–1.26	1.33–1.49	–
Lin [250]	$10^{-3}$	1.08	–	1.24	1.14
Zhou [251]	$10^{-3}$	1.17	1.25–1.29	1.31–1.37	–
Zhou [252]	$10^{-3}$–$10^{-4}$	1.26–1.39	1.28–1.42	1.29–1.49	1.24–1.45
Xiao [206]	$10^{-5}$–$10^{-2}$	1.00–1.21	0.91–1.10	0.93–1.10	0.93–1.10
Wu [47]	$10^{-2}$	1.26	1.46	1.27–1.35	–
Chen [54]	$10^{-2}$	1.17	1.35	1.35	–
Wang [253]	$10^{-3}$–$10^{-2.3}$	1.28–1.47	1.30	1.33	–
Yu [175]	$10^{-5}$–$10^{-2}$	1.00–1.36	1.00–1.36	0.84–1.06	–
Cyclic					
Zhou [251]	1 Hz	1.17	1.25	1.31	–
Chen [54]	5 Hz	1.17	1.35	1.35	–
Zhou [252]	1 Hz	1.26	1.28	1.29	1.24
Wu [259]	1–5 Hz	–	1.04–1.19	–	–
Lin [250]	0.5–2 Hz	1.13	–	1.24–1.28	1.13–1.24

**Table 5:** DIF in tension for large-aggregate concrete.

Reference	DIF (Dry)	DIF (Wet)	Strain Rate s^−1^	Load History (Preload)	Tension Test	Concrete Type	NMSA (mm)
[257]	1.1	1.3	$10^{-3}$–$10^{-2}$	Precycled	Stress Reversal and Direct Tension	Mass	75
[2]	1.31–1.83	–	$10^{-3}$	–	Direct and Split Tension	Dam cores	38–127
[256]	1.07–1.75	–	$10^{-4}$–$10^{-2}$	Compression (72%) and compression cycles (38–57%)	Direct	Fully graded	80–120
[285]	1.06–1.73	1.58–1.61	$10^{-4}$–$10^{-2}$	–	Split	Dam cores	75–150
[249]	1.23–1.49		$10^{-3}$–$10^{-2}$	Tension (40–80%)	Flexural	Fully graded and wet-screened	40–150
[290]	1.22	–	$10^{-4}$–$10^{-3}$	–	Flexural	Fully graded	40–80
[251]	1.17–1.37	–	$10^{-4}$–$10^{-3}$	Tension (40–80%)	Flexural	Three-graded	40–80
[252]	1.24–1.49	–	$10^{-4}$–$10^{-3}$	Tension (40–90%)	Flexural and cyclic	Wet-screened	40
[291]	1.13–1.17	–	$10^{-3}$	–	Flexural	Three-graded	40–80
[254]	1.1–1.45	–	$10^{-6}$–$10^{-2}$	–	Flexural, direct and split	Mass	40
[47]	1.14–1.46	1.15–1.32	$10^{-6}$–$10^{-3}$	–	Split	Mass	40–150
[287]	1.18–1.59	–	$10^{-4}$–$10^{-2}$	–	Direct	Three-graded and wet-screened	80
[53]	1.13–1.46	–	$10^{-6}$–$10^{-4}$	–	Split	Mass	40
[284]	1.18–1.59	–	$10^{-4}$–$10^{-2}$	–	Direct	Fully graded and wet-screened	80
[54]	1.17–1.80	–	$10^{-2}$	Tension (40–80%)	Flexural, monotonic and cyclic	Fully graded	150
[294]	1.18–1.59	–	$10^{-4}$–$10^{-2}$	–	Direct	Mass	80
[288]	1.18–1.59	–	$10^{-4}$–$10^{-2}$	–	Direct	Fully graded and wet-screened	80
[253]	1.28–1.47	–	$10^{-3}$	Tension (30–60%)	Direct	Dam cores	80
[292]	1.12–1.30	1.08–1.19	$10^{-4}$–$10^{-2}$	Freeze–thaw cycles	Direct	Hydraulic concrete	80
[289]	1.15–1.56	–	$10^{-4}$	–	Direct tension and cyclic	Fully graded and wet-screened	80
[293]	1.11–1.30	–	$10^{-4}$–$10^{-2}$	–	Direct	Mass	80
[295]	1.09–1.32	–	$10^{-4}$–$10^{-3}$	–	Split	Dam cores	150
[176]	1.58	–	$10^{-3}$	–	Direct	Mass	40–80
[286]	1.27–1.39	–	$10^{-4}$–$10^{-3}$	–	Flexural	Fully graded and wet-screened	150

**Table 6:** Tests performed at high strain rates, grouped by concrete type.

Reference	Method	Test Type	Material	Parameter	Strain Rate (1/s)	DIF
Ordinary-Strength Concrete (OSC)						
[18]	AFP	Spall	OSC	Tensile strength	20	6
[19]	AFP	Spall	OSC	Tensile strength	23	6
[179]	SHPB	Direct	OSC	Tensile strength	0.5	1.75
[300]	SHPB	Split	OSC	Tensile strength	0.9	3.6
[300]	SHPB	Direct	OSC	Tensile strength	0.65	2.6
[301]	SHPB	Direct	OSC	Tensile strength	0.3	1.53
[165]	SHPB	Split	OSC	Tensile strength	7.7	3.6
[28]	SHPB	Split	OSC	Tensile strength	8	3.0
[31]	SHPB	Spall	OSC	Tensile strength	100	4.0
[31]	SHPB	Split	OSC	Tensile strength	100	4.0
[29]	SHPB	Direct	OSC	Tensile strength	10	3.5
[302]	SHPB	Direct	OSC	Tensile strength	10	3.7
[303]	SHPB	Spall	OSC	Tensile strength	53	4.8
[39]	SHPB	Direct	OSC	Tensile strength	70	3.93
[304]	SHPB	Spall	OSC	Tensile strength	100	2.2
[46]	SHPB	Spall	OSC	tensile strength	100	3.8
[51]	SHPB	Flexural	OSC	Tensile strength	67	8.1
[305]	SHPB	Direct	OSC	Tensile strength	150	2.0
[50]	MSHPB	Direct	OSC	Tensile strength	12	3.5
[56]	SHPB	Split	OSC	Tensile strength	100	3.9
[79]	SHPB	Split	OSC	Tensile strength	7.6	2.87
High-Strength/-Performance Concrete (HSC/HPC)						
[38]	SHPB	Spall	HPC	Tensile strength	29	3.3
[306]	SHPB	Spall	HPC	Tensile strength	160	3.8
[74]	SHPB	Split	HSC	Tensile strength	0.8	2.2
[73]	SHPB	Split	HSC	Tensile strength	6.6	4.5
[78]	SHPB	Split	Wet HSC	Tensile strength	14	4.8
[78]	SHPB	Split	Dry HSC	Tensile strength	14	4.8
[79]	SHPB	Split	HSC	Tensile strength	7.9	2.6
Reactive Powder and Ultra-High-Performance Concrete (RPC/UHPC/UHSC)						
[37]	Servo-hydraulic	Direct	RPC	Tensile strength	50	1.7
[171]	CAGBBD	Flexural	UHPC	Tensile strength	10	3.6
[45]	Drop weight	Flexural	UHPC	tensile strength	1.7	1.5
[49]	SHPB	Spall	UHPC	Tensile strength	66	6.7
[52]	SHPB	Spall	UHPC	Tensile strength	125	4.8
[57]	SEFIM	Direct	UHPC	Tensile strength	11	2.4
[59]	Servo-hydraulic	Direct	UHPC	Tensile strength	0.1	1.2
[58]	Servo-hydraulic	Direct	UHPC	Tensile strength	10	1.4
[307]	SHPB	Spall	UHSC	Tensile strength	80	3.2
[65]	SHPB	Direct	UHPC	Tensile strength	420	3.6
[66]	SEFIM	Direct	UHPC	Tensile strength	26	1.7
[63]	SEFIM	Direct	UHPC	Tensile strength	120	3.4
[60]	SHPB	Direct	UHPC	Tensile strength	100	1.8
[61]	SHPB	Direct	UHPC	Tensile strength	300	2.4
[67]	Servo-hydraulic	Direct	UHPC	Tensile strength	0.1	1.2
[69]	SEFIM	Direct	UHPC	Tensile strength	26	2.6
[68]	SEFIM	Direct	UHPC	Tensile strength	125	2.6
Fiber-Reinforced and Strain-Hardening Concretes (HPRFC, UHPRFC, SHCC)						
[22]	Explosive	Spall	RFC	Tensile strength	157	7.0
[306]	SHPB	Spall	HPRFC	Tensile strength	160	5.3
[306]	SHPB	Spall	SHCC	Tensile strength	160	5.8
[49]	SHPB	Spall	UHPRFC	Tensile strength	56	3.9
[305]	SHPB	Direct	HPRFC	Tensile strength	150	2.6
[44]	SHPB	Spall	UHPRFC	Tensile strength	128	4.89
Moisture-Conditioned Concrete (Dry vs. Wet)						
[24]	SHPB	Direct	Wet OSC	Tensile strength	1	2.4
[24]	SHPB	Direct	Wet OSC	Elastic modulus	1	1.2
[163]	Shock tube	Slab	Dry OSC	Tensile strength	1	1.5
[163]	Shock tube	Slab	Wet OSC	Tensile strength	1	2.0
[28]	SHPB	Split	Dry OSC	Tensile strength	6	3.6
[28]	SHPB	Split	Wet OSC	Tensile strength	6	4.0
[5]	SHPB	Spall	Dry OSC	Tensile strength	100	8.0
[5]	SHPB	Spall	Wet OSC	Tensile strength	128	13.0
[6]	SHPB	Spall	Wet OSC	Tensile strength	150	8.0
[6]	SHPB	Spall	Dry OSC	Tensile strength	150	5.0
[78]	SHPB	Split	Wet OSC	Tensile strength	14	6.0
[78]	SHPB	Split	Dry OSC	Tensile strength	14	5.0

**Table 7:** Numerical modeling studies on dynamic tensile behavior of concrete.

Reference	Numerical Method	Key Focus
[83]	3D FEM Cohesive surface elements	Dynamic fracture Brazilian tests Exploring size effects
[30]	2D DEM Voronoï-based particle meshing Delaunay lattice bonding	Dynamic spalling and fracture in concrete
[84]	2D FEM Isotropic local damage model Viscoplastic damage model	Modified SHB test fracture behavior under ultra-high loading rates
[35]	FEM LS-DYNA Continuum damage accumulation model	Dynamic tensile failure (spalling)
[85]	Mesoscale FEM Dynamic damage model	Dynamic bending strength of large fully graded beams Influence of load history
[86]	Mesoscopic FEM Anisotropic damage model	Dynamic tensile behavior in SHPB/spalling tests Influence of water content
[87]	3D mesoscale FEM	Dynamic spalling and fracture in concrete
[49]	FEM LS-DYNA JHC model	Dynamic tensile behavior of UHPCC
[88]	FEM ABAQUS DFH model	Dynamic spalling and fracture in concrete
[53]	Thermodynamic model	Dynamic splitting tensile strength of concrete
[89]	Mesoscale FEM AUTODYN Drucker–Prager model	Lateral inertia confinement and specimen size influence
[90]	FEM LS-DYNA	Dynamic tensile behavior of cement mortar
[91]	2D mesostructural FEM	Influence of aggregate size, distribution, and ITZ properties
[92]	Mesoscale FEM Random aggregate model	Dynamic bending strength Material heterogeneity Influence of load history and aggregate grading
[93]	3D FEM Microplane model	Strain rate vs. structural inertia
[94]	2D mesoscopic FEM VEVPD model	Water content and ITZ strength SHB and MSHP tests
[95]	2D mesoscale FEM ABAQUS CDP model	Mesostructural heterogeneity
[96]	FEM ABAQUS DFH-KST coupled model	Influence of free water content on concrete’s ballistic resistance
[97]	Bond-based peridynamics Fortran90 Prototype microelastic brittle	Dynamic failure of concrete Brazilian discs in SHPB tests
[98]	Partially saturated FEM Barton’s empirical model	Influence of water pressure and crack permeability during crack growth in concrete dams
[99]	FEM ABAQUS Three-parameter damage model	Dynamic tensile failure of brittle materials
[100]	Mesoscale FEM Random aggregate model Occupation and removal method	Flexural failure behavior of fully graded concrete for dams with realistic aggregate shapes and gradations Influence of load history
[101]	2D mesoscale FEM ABAQUS Maxwell-type rheological model	Influence of ITZ properties, aggregate content, and porosity
[102]	FEM ABAQUS Drucker–Prager model	Influence of the inertial effect
[103]	3D mesoscale FEM LS-DYNA	Dynamic tensile behavior of UHPC using explicitly modeled fiber reinforcement
[104]	FEM ABAQUS Concrete damaged plasticity	Influence of the inertial effect
[105]	3D FEM LS-DYNA Johnson–Holmquist concrete	Dynamic Brazilian tests in SHPB apparatus
[106]	FEM LS-DYNA Comparison of models	Blast loads, missile penetration, and three-point bending
[107]	3D FEM ABAQUS	Comparing mortar vs. concrete dynamic behavior
[108]	3D mesoscale FEM LS-DYNA Karagozian and Case model	Developing a high-fidelity 3D mesoscale concrete model Influence of ITZ and aggregate size distribution
[109]	2D XFEM (ABAQUS)	Dynamic tensile strength and elastic modulus of UHPC from Brazilian tests
[110]	FEM LS-DYNA Holmquist–Johnson–Cook model	Comparing numerical results (inertia only) vs. SHPB experiments (inertia + strain rate)
[111]	Finite weakest link model	Influence of strain rate on size effect and cracking
[112]	3D FEM ABAQUS Drucker–Prager model	Strain rate vs. inertial effects
[113]	Mesoscale FEM Random aggregate model	Influence of load history
[114]	3D Mesoscopic FEM Concrete damaged plasticity model	Size and inertial effects
[115]	FEM ABAQUS Random aggregate model Concrete damage plasticity	Influence of aggregate volume fraction and beam depth
[116]	2D FEM with random aggregate model	Dynamic tensile failure and size effect in concrete
[117]	3D FEM ABAQUS Rheological traction separation model	Dynamic crack initiation and propagation under spall tests
[118]	3D DEM Particle flow code	Concrete strength, modulus, crack pattern, and damage evolution
[119]	Bond-based peridynamic model	Fracture behavior of coal under SHPB loading
[120]	FEM ABAQUS Drucker–Prager model	Isolating and quantifying the inertial effect
[121]	Base force element method	Mesoscale dynamic behavior of recycled aggregate concrete
[122]	3D mesoscopic FEM model	Meso-level failure mechanisms of concrete under dynamic splitting tension
[123]	Mesoscale 2D discrete element method	Nonlinear effect of pore water
[3]	2D mesoscale bonded particle model Particle Flow Code	Size, inertia, and multiple crack effects
[124]	Modified Intermediately Homogenized Peridynamic Ordinary State-Based Peridynamics Framework	Influence of water content
[125]	3D bonded particle model LS-DYNA	Influence of grain heterogeneity and cement interface strength
[126]	3D stochastic mesoscale FEM model LS-DYNA	Dynamic splitting tensile behavior of UHP-HFRC under SHPB loading
[127]	FEM LS-DYNA Karagozian and Case model	Influence of water content
[128]	FEM ABAQUS Thermal–mechanical sequential coupled mesoscale	Influence of water content, temperature, and size effect
[129]	DEM-CFD YADE Fully coupled hydro-mechnical model	Influence of water content on strength and fracture
[130]	2D and 3D mesoscale FEM models	Dynamic tensile behavior of multi-scale self-compacting concrete
[131]	3D FEM LS-DYNA	Dynamic response of FRP-reinforced UHPC beams under impact
[132]	2D DEM Parallel bonded model	Propose and validate a new specimen design for direct tensile testing of concrete using concentric cylindrical cuts from a cube
[133]	FEM ANSYS/LS-DYNA Holmquist–Johnson–Cook model	Influence of matrix strength
[134]	FEM ABAQUS	Dynamic crack propagation in mortar–granite interfaces
[135]	FEM ABAQUS Johnson–Holmquist–Cook model	Influence of steel fiber volume on spalling strength
[136]	Coupled FDM-DEM PFC3D FLAC3D Parallel bond model	Dynamic splitting behavior of coral aggregate seawater shotcrete under SHPB
[137]	Coupled FDM-DEM Particle Flow Code	Dynamic fracture behavior of bimaterial rock–concrete Brazilian disks with a central interface crack under SHPB loading
[138]	FEM ABAQUS Cohesive element model	Influence of pore structure on concrete’s impact tensile behavior

**Table 8:** DIFs in the guidelines for concrete dams.

Year	Guideline	Agency	Reference	DIF			
				$f_{t}$	$f_{c}^{\prime }$	E	ν
1976	Design of gravity dams	USBR	[261]	1	1	1	1
1977	Design Criteria for Concrete Arch and Gravity Dam	USBR	[262]	1	1	1	1
1977	Design of arch dams	USBR	[263]	1	1	1	1
1987	Design of small dams	USBR	[264]	1.7	1	1.2	1
1994	Arch dam design	USACE	[265]	1.3	1.3	1.2	1.25
1995	Gravity dam design	USACE	[266]	1.5	1	1	1
1999	Engineering guidelines for evaluation of hydropower projects— Chapter 11 Arch Dams	FERC	[267]	1.5	1.3	1.25	1
2003	Guide pour l’évaluation de la sécurité sismique	Hydro-Quebec	[268]	1.5	1.25	1.25	1.25
2006	State-of-Practice for the Nonlinear Analysis of Concrete Dams	USBR	[269]	1.5	1.2	1.5	1
2007	Earthquake Design and Evaluation of Concrete Hydraulic Structures	USACE	[270]	1.5	1.15	1.15	0.7
2007	Standard Specifications for Concrete Structures— Dam concrete	JSCE	[271]	1.3	1.3	1	1
2009	The Physical Properties of Hardened Conventional Concrete in Dams	ICOLD	[272]	1.35	1	1	1
2013	Design of Double Curvature Arch Dams	USBR	[273]	1	1	1	1
2016	Engineering guidelines for evaluation of hydropower projects— Chapter 3 Gravity Dams.	FERC	[274]	1	1	1	1
2016	Directive relative à la sécurité des ouvrages d’accumulation Partie C3: sécurité aux séismes	OFEN	[275]	1.3	1.3	1.25	1
2016	Guideline for FE analyses of concrete dams	SERC	[276]	1.5	1	1.25	1
2017	Guidelines for Design of Dams and Appurtenant Structures for Earthquake	ANCOLD	[277]	1.5	1	1.25	1

## Data Availability

No new data were created or analyzed in this study. Data sharing is not applicable to this article.

## Abbreviations

The following abbreviations are used in this manuscript:

ANCOLD	Australian National Committee on Large Dams
AFP	Air-Fired Projectile
BPM	Bonded Particle Model
CAGBBD	Compressed Air Gun Block Bar Device
CEB-FIP	Comité Euro-international du béton and Fédération Internationale de la Précontrainte
CZM	Cohesive Zone Model
DEM	Discrete Element Method
DIF	Dynamic Increase Factor
EMS	Equivalent Momentum Scheme
FEM	Finite Element Method
FERC	Federal Energy Regulatory Commission
FRC	Fiber-Reinforced Concrete
FDM	Finite Difference Method
HPC	High-Performance Concrete
HJC	Holmquist–Johnson–Cook
HSC	High-Strength Concrete
HSV	High Stress Volume
JSCE	Japan Society of Civil Engineers
K&C	Karagozian and Case
K&I	Kamran and Iqbal

MSHPB	Modified Split Hopkinson Pressure Bar
NRC	National Research Council
NMSA	Nominal Maximum Size Aggregate
OFEN	Office Fédéral de l’Énergie
OSC	Ordinary-Strength Concrete
PD	Peridynamics
RBSM	Rigid-Body Spring Model
RFC	Reinforced Fiber Concrete
RPC	Reactive Powder Concrete
SEFIM	Strain Energy Frame Impact Machine
SERC	Swedish Energy Research Centre
SHCC	Strain Hardening Cement-Based Composite
SHPB	Split Hopkinson Pressure Bar
USACE	United States Army Corps of Engineers
USBR	United States Department of the Interior—Bureau of Reclamation
UPHC	Ultra-High-Performance Concrete
UHSC	Ultra-High-Strength Concrete
UHPGC	Ultra-High-Performance Geopolymer Concrete
UHTCC	Ultra-High-Toughness Cementitious Composite
